# Spinal cords: Symphonies of interneurons across species

**DOI:** 10.3389/fncir.2023.1146449

**Published:** 2023-04-26

**Authors:** Alexia C. Wilson, Lora B. Sweeney

**Affiliations:** Institute of Science and Technology Austria (IST Austria), Klosterneuburg, Lower Austria, Austria

**Keywords:** spinal cord, interneuron, motor control, vertebrate evolution, movement, cross-species comparison

## Abstract

Vertebrate movement is orchestrated by spinal inter- and motor neurons that, together with sensory and cognitive input, produce dynamic motor behaviors. These behaviors vary from the simple undulatory swimming of fish and larval aquatic species to the highly coordinated running, reaching and grasping of mice, humans and other mammals. This variation raises the fundamental question of how spinal circuits have changed in register with motor behavior. In simple, undulatory fish, exemplified by the lamprey, two broad classes of interneurons shape motor neuron output: ipsilateral-projecting excitatory neurons, and commissural-projecting inhibitory neurons. An additional class of ipsilateral inhibitory neurons is required to generate escape swim behavior in larval zebrafish and tadpoles. In limbed vertebrates, a more complex spinal neuron composition is observed. In this review, we provide evidence that movement elaboration correlates with an increase and specialization of these three basic interneuron types into molecularly, anatomically, and functionally distinct subpopulations. We summarize recent work linking neuron types to movement-pattern generation across fish, amphibians, reptiles, birds and mammals.

## Introduction

**Figure 1:**
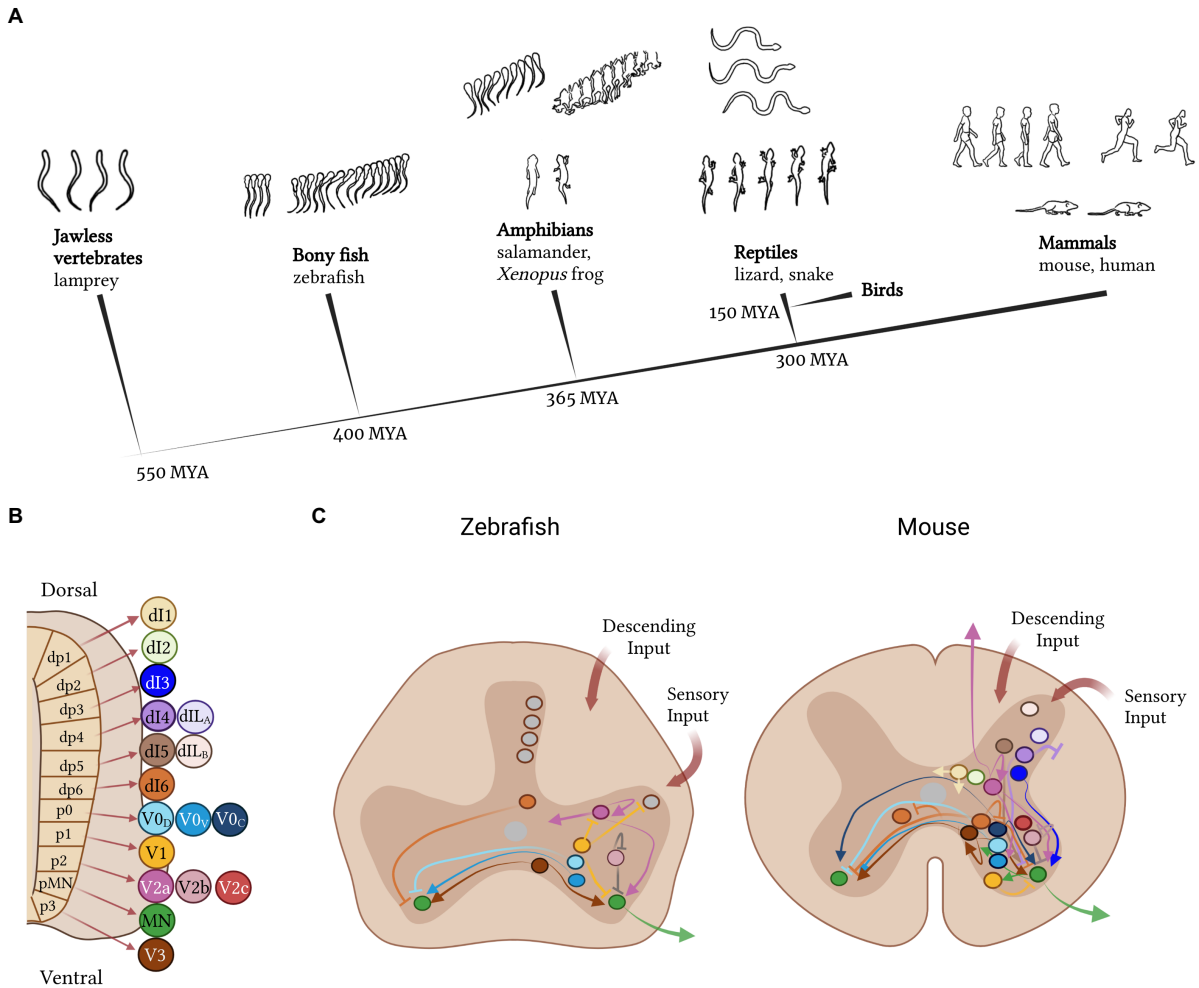
A cross-species comparison of the neural basis of vertebrate movement. **(A)** Cladogram of vertebrate evolution with illustrations of movement patterns for each of the species listed as examples. The lamprey is the most primitive vertebrate and exhibits simple, undulatory swimming; zebrafish display more complex swimming patterns; the frog and salamander use both tail and limbs for movement; reptiles exhibit diagonal limb coordination; and mammals display complex fore−/hindlimb gaits. **(B)** Cardinal neuron classes that make up the spinal cord circuitry are derived from 11 progenitor domains. Some domains give rise to more than one neuron class, e.g., the p2 domain gives rise to the V2a, V2b, and V2c interneurons. **(C)** Comparison of interneuron subtypes and projection patterns in the spinal cord of zebrafish versus mice. Colors represent different neuron classes; gray represents neurons without a clear cardinal class identity.

Vertebrates exhibit a wide range of movement patterns. Across species and evolutionary time, they have transitioned from axial-based swimming to limb-based locomotion. Between species, they have uniquely adapted their movement repertoires to their environment, physiological needs, and mode of locomotion (Figure 1A; Hill et al., 2016; Sillar et al., 2016; Auclair et al., 2020). Fish, for example, rely on precise and alternating contraction of segments along the rostrocaudal axis to generate slow, undulatory swimming. Mice coordinate the flexor and extensor muscles of the limb to grasp food pellets, run on a wheel, swim, and perform stereotyped repetitive grooming behaviors. Frogs adopt fish-like undulatory movement as tadpoles, transition to limb-based locomotion during metamorphosis, and as adults, predominantly rely on synchronous limb movement (Combes et al., 2004; Rauscent et al., 2006; Roberts et al., 2010; Bacqué-Cazenave et al., 2018; Currie and Sillar, 2018). This contrasts with other amphibians, such as salamanders, which maintain both undulatory tail and alternating limb movement throughout life. Like salamander, limbed reptiles, and most mammals, including mice and humans, similarly alternate their limb muscles at all speeds as a default behavior (Grillner, 2011; Rossignol, 2011; Kiehn, 2016). Notable exceptions to this are snakes, which have lost their limbs and exhibit only axial body movement. These many differences in movement between species raise the question of how their underlying motor circuits differ.

### Spinal circuit form and function

Over a century ago, Sherrington and Brown demonstrated that motor circuits of the spinal cord were the core of movement generation. Sherrington highlighted the integrative nature of spinal, sensory and central circuits in reciprocal motor action in the cat (Sherrington, 1906). Brown then proposed the half-center model, in which rhythm is generated by two half-centers in the spinal cord that reciprocally inhibit each other (Brown, 1911). Together, these two half centers, and their constituent spinal circuits, were dubbed central pattern generators (CPGs). Experimental evidence for such a CPG organization and initial characterization of spinal reflex behaviors was first described in invertebrates, and then, in the spinalized cat (Wilson and Wyman, 1965; Forssberg, 1979; Forssberg et al., 1980a,b; Lovely et al., 1990; Rossignol et al., 2006; Stuart and Hultborn, 2008). Although evolutionarily distant, in both, “normal” locomotor patterns with appropriate excitation were present even in the absence of descending input, supporting that rhythm-generating modules were intrinsic to the spinal cord. Later studies in the lamprey and *Xenopus* tadpole revealed that ipsilateral excitatory drive combined with reciprocal inhibition made up the core architecture of the vertebrate CPG (Forssberg et al., 1980b; Buchanan and Grillner, 1987; Roberts et al., 2010).

Since Sherrington and Brown, our understanding of spinal circuits has rapidly advanced due to the development of genetic approaches for identifying and manipulating neurons and physiological tools for recording, activating, or suppressing them (Luo et al., 2008). It is still believed that CPGs in the spinal cord, consisting of motor neurons and interneurons, are the modules responsible for transforming constant input into rhythmic output. However, we now understand that each unit of the CPG is composed of multiple neuronal subtypes (Grillner and El Manira, 2020).

Motor neurons, the best-characterized example of this subdivision, form molecularly- and anatomically-distinct columns, divisions and pools based on the body region and muscle they target (Dasen, 2022). This specificity is dictated by a single family of transcription factors, the *Hox* genes (Tsuchida et al., 1994; Arber et al., 1999; Dasen and Jessell, 2009; Philippidou and Dasen, 2013). Motor columns divide into motor pools which are further partitioned into alpha, beta, and gamma subtypes based on their fiber versus spindle innervation pattern (Dasen, 2022). The alpha subclass is further subdivided into fast-fatigue, fast-fatigue resistant or slow types based on the specific fiber type they innervate (Friese et al., 2009; Alkaslasi et al., 2021). The sequential and coordinated activation of these motor neuron types by a network of excitatory and inhibitory interneurons underlies coordinated movement.

Like motor neurons, excitatory and inhibitory interneurons in the spinal cord, can be similarly compartmentalized by their molecular, anatomical and functional properties (Briscoe et al., 2000; Jessell, 2000; Sengupta and Bagnall, 2023). They subdivide into at least 11 classes based on their developmental origin, gene expression and anatomical projection pattern: six dorsal classes (dI1-6), and five ventral classes (V0, V1, V2a, V2b, and V3; Goulding, 2009; Figure 1B). From a molecular perspective, recent single-cell sequencing work in the developing and adult mouse spinal cord has suggested that these eleven classes can be split into further numerous molecularly distinct cell types (Häring et al., 2018; Delile et al., 2019; Blum et al., 2021; Russ et al., 2021). Birth date, projection range, and motor/sensory function divides them even more (Osseward et al., 2021). Even a single class, such as V1, can contain up to 50 distinct subpopulations (Francius et al., 2013; Bikoff et al., 2016; Gabitto et al., 2016; Sweeney et al., 2018). From a physiological perspective, the response properties of interneurons also segregate them, exemplified by the recruitment of distinct excitatory V2a subtypes at slow or fast locomotor speeds (Zhong et al., 2011; Ampatzis et al., 2014). This demonstrates a broad organization of interneurons into cardinal classes and yet, a precise subdivision of the neurons within these classes based on their molecular and physiological characteristics.

### Cross-species approach to study spinal circuit scaling

This large amount of spinal neuron subtype heterogeneity could provide the link between specialized vertebrate movements and their underlying spinal circuits. Many recent studies have sought to test this possibility using species with diverse motor outputs and high levels of genetic access, such as the zebrafish and mouse (Gosgnach et al., 2017; Grillner and El Manira, 2020).

Here, we aim to lay the foundation for a complementary cross-species approach. Such an approach could differentiate cell types required for swimming (lamprey, fish, tadpoles) versus limb movement (frog, mice, humans, horses), or distinct movement capabilities between closely related species, such as rodents that hop versus run (kangaroo rat versus mouse) or mammals with varying gaits (mouse, horse, human). These approaches have the potential to pinpoint shared versus species-specific neural components of movement, taking us one step closer to determining how they correspond.

Such shared components include the precise coordination of muscle groups along the rostrocaudal, dorsoventral, and left–right body, and body-part axes; the variation of movement in a speed-dependent manner; and the ability of increasing drive to recruit additional motor units sequentially (Henneman et al., 1965; Gustafsson and Pinter, 1984a,b; Cope and Pinter, 1995; McLean et al., 2007; Gabriel et al., 2011). This graded recruitment enables smooth transitions from slower or weaker to faster or stronger movements. In addition, reflex coordination has a modular organization, exemplified by studies in the frog, in which locomotion can be fractionated into motor primitives for each reflex (Mussa-Ivaldi et al., 1994; Hart and Giszter, 2004). This principle is likely to extend across limbed species (Levine et al., 2014). Finally, for an organism to survive in its environment, it must also integrate sensory information and vary the type, amplitude and speed of its movement accordingly (Zanker, 2010).

Many components of movement however are species-specific, with one of the best examples being the speed-dependent expression of gaits. In many tetrapods, faster locomotion is achieved by gait transitions: walking at slow speeds, trotting at intermediate speeds and galloping at high speeds (Biewener, 2006; Reilly et al., 2007; Fu et al., 2021). Horses exemplify this: the phase relationship of their limbs relative to each other varies between each of their speed-dependent gaits. A species-specific molecular mechanism has even been identified for this phase relationship with a mutation in the *Dmrt3* gene resulting in the misspecification of a dorsal interneuron population and the appearance of either unnatural or additional gaits (Andersson et al., 2012). Additionally, in limbed vertebrates, spinal cord composition varies across the rostro-caudal axis (Dasen and Jessell, 2009; Francius et al., 2013; Sweeney et al., 2018; Dasen, 2022). In this review, we largely focus on limb levels when discussing spinal cord architecture in limbed species.

Mechanistically, these shared and specific features between vertebrate species raise several fundamental questions that this review aims to explore. Is cell type heterogeneity in the spinal cord a correlate of movement diversity? At what level – molecular, anatomical and/or physiological – do cell types converge or diverge across species, and to what extent do these properties correspond? How do conserved features of movement, such as left–right coordination, map onto spinal cord cell types? And how do these maps vary for divergent features, such as gaits? Moreover, given the variation in sensory and cognitive inputs between species, do spinal circuits similarly vary and if yes, for which cell types and on what level?

There has never been a better time to make such cross-species comparisons. Single-cell sequencing has enabled detailed molecular comparisons of neuronal classes in the spinal cord within and across species (Häring et al., 2018; Sathyamurthy et al., 2018; Delile et al., 2019; Shafer, 2019; Russ et al., 2021). It is now possible to record hundreds of neurons in an actively-moving animal, empowering us to validate and extend findings which previously could only be made in an isolated spinal cord (Long and Lee, 2012; Lang et al., 2021). We can also now take advantage of the vast anatomical and physiological knowledge of spinal neuron function in simpler organisms, in which they have been more comprehensively studied (Grillner and El Manira, 2020), to make novel predictions about their role in more complex ones.

### Cardinal classes across vertebrates

In this review, we provide arguments to support the hypothesis that, as you move from simple swimming to limb-based movement across vertebrate evolution, spinal interneurons are compartmentalized into distinct molecular, anatomical and functional subclasses. Although these changes in spinal circuitry are accompanied by parallel changes in higher brain centers (Northcutt, 2002; Gonzalez-Voyer et al., 2009; Karten, 2015; Leiras et al., 2022), these topics will not be discussed in this review. Here, we focus on interneurons in the spinal cord, structuring our discussion using the cardinal class organization of mammals, which captures both molecular and functional properties of each neuron class (Delile et al., 2019). We start with the ventral excitatory classes: ipsilaterally-projecting V2a neurons and bilaterally-projecting V3 neurons. We then describe the mixed excitatory and inhibitory commissural class of V0 neurons and the inhibitory ipsilateral V1 and V2b neurons. Finally, we end with the dorsal inhibitory dI6 and mixed dI1-5 neurons, of which the least amount is known. In each section, we summarize our current knowledge of the conservation and divergence of cell type architecture across vertebrate species, focusing on zebrafish and mouse and, when possible, providing examples from lesser-studied species such as turtle and chicken.

## V2a excitatory neurons

Ipsilaterally-projecting V2a excitatory neurons arise from the p2 progenitor domain and are defined by the transcription factor VSX2 in zebrafish and mice (Barabino et al., 1997; Thaler et al., 1999). During development, p2 progenitors differentiate into at least two subpopulations: V2a excitatory neurons, discussed here, and V2b inhibitory neurons, marked by GATA2/3 expression and discussed below (Karunaratne et al., 2002; Al-Mosawie et al., 2007; Lundfald et al., 2007; Peng et al., 2007). In zebrafish, where interneurons are often named by their projection patterns, these neurons correspond to the circumferential descending (CiD) cells (Hale et al., 2001; Kimura et al., 2006). Glutamate is a key neurotransmitter employed by V2a excitatory neurons in all species, with expression of the vesicular glutamate transporter 2 and blockage of V2a-derived motor neuron EPSPs by glutamate receptor antagonists, shared properties of zebrafish and mice (Kimura et al., 2006; Crone et al., 2008; Ampatzis et al., 2014).

In the lamprey, ipsilateral excitatory neurons provide the drive for the locomotor network (Dale, 1986; Buchanan and Grillner, 1987; Buchanan and McPherson, 1995; Fernández-López et al., 2012). Although it is unknown whether they express Vsx2, their connectivity pattern and functional role as the drivers of movement suggest they may represent a primitive V2a population. In the lamprey, tadpole, zebrafish and mouse, this group of neurons receives descending and peripheral sensory input, and excites other V2a interneurons, commissural interneurons and motor neurons (Dale, 1986; Buchanan and Grillner, 1987; Buchanan et al., 1989; Ohta and Grillner, 1989; Parker and Grillner, 2000; Kimura et al., 2006, 2013; Li et al., 2006, 2009; Crone et al., 2008; Soffe et al., 2009; Dougherty and Kiehn, 2010; Ampatzis et al., 2014; Hayashi et al., 2018; Li and Soffe, 2019; Menelaou and McLean, 2019). Recurrent connections between V2a neurons generate consistency in motor output (Buchanan and Grillner, 1987; Buchanan et al., 1989; Cangiano and Grillner, 2005; Hayashi et al., 2018; Menelaou and McLean, 2019) and connections between V2a and commissural neurons implicate the V2a population in the coordination of the left and right side (Buchanan and Grillner, 1987; Crone et al., 2008; Dougherty and Kiehn, 2010; Menelaou and McLean, 2019). Recent studies detailing how V2a neurons drive tail and limb movement patterns in zebrafish and mouse, respectively, provide a framework to understand how molecular, anatomical and functional subtypes correspond and scale with movement complexity.

### Zebrafish V2a neurons

In zebrafish, V2a excitatory neurons are both necessary and sufficient to induce a normal swim pattern. Supporting this, action potentials in V2a neurons usually occur before those in motor neurons (Ampatzis et al., 2014), and optogenetic activation of this class generates swimming (Eklöf Ljunggren et al., 2014), implicating them as drivers of the swim circuit. V2a neurons are also present in the hindbrain, where optogenetic activation drives, while inactivation impairs or stops, swimming (Kimura et al., 2013). Acute and selective ablation of V2a neurons has three potent effects on swimming activity: an increase in the threshold for its initiation, a decrease in locomotor-related burst frequency, and a change in the rostrocaudal propagation of activity (Eklöf-Ljunggren et al., 2012). Similar changes were seen when swimming was induced by electrical stimulation or NMDA application, suggesting they are due to perturbations in the excitability of the swim circuit (Eklöf-Ljunggren et al., 2012). This experimental evidence provides strong support that V2a neurons are crucial drivers and determinants of locomotion in zebrafish.

It was also observed in zebrafish that specific V2a subpopulations are recruited in a speed-dependent manner. As the fish’s swim speed increased, ventrally-located V2a neurons were recruited before dorsally-located ones (Kimura et al., 2006; McLean et al., 2008; McLean and Fetcho, 2009; Ausborn et al., 2012). Selective ablation of dorsal V2a neurons decreased peak, without altering sustained, swim frequency; whereas optogenetic activation of the entire V2a population recruited ventral, but not dorsal, subpopulations (Eklöf-Ljunggren et al., 2012). This suggested a speed-dependence of V2a excitatory subpopulations in tail locomotion, dictated by their dorsoventral location and recruitment-threshold.

**Figure 2:**
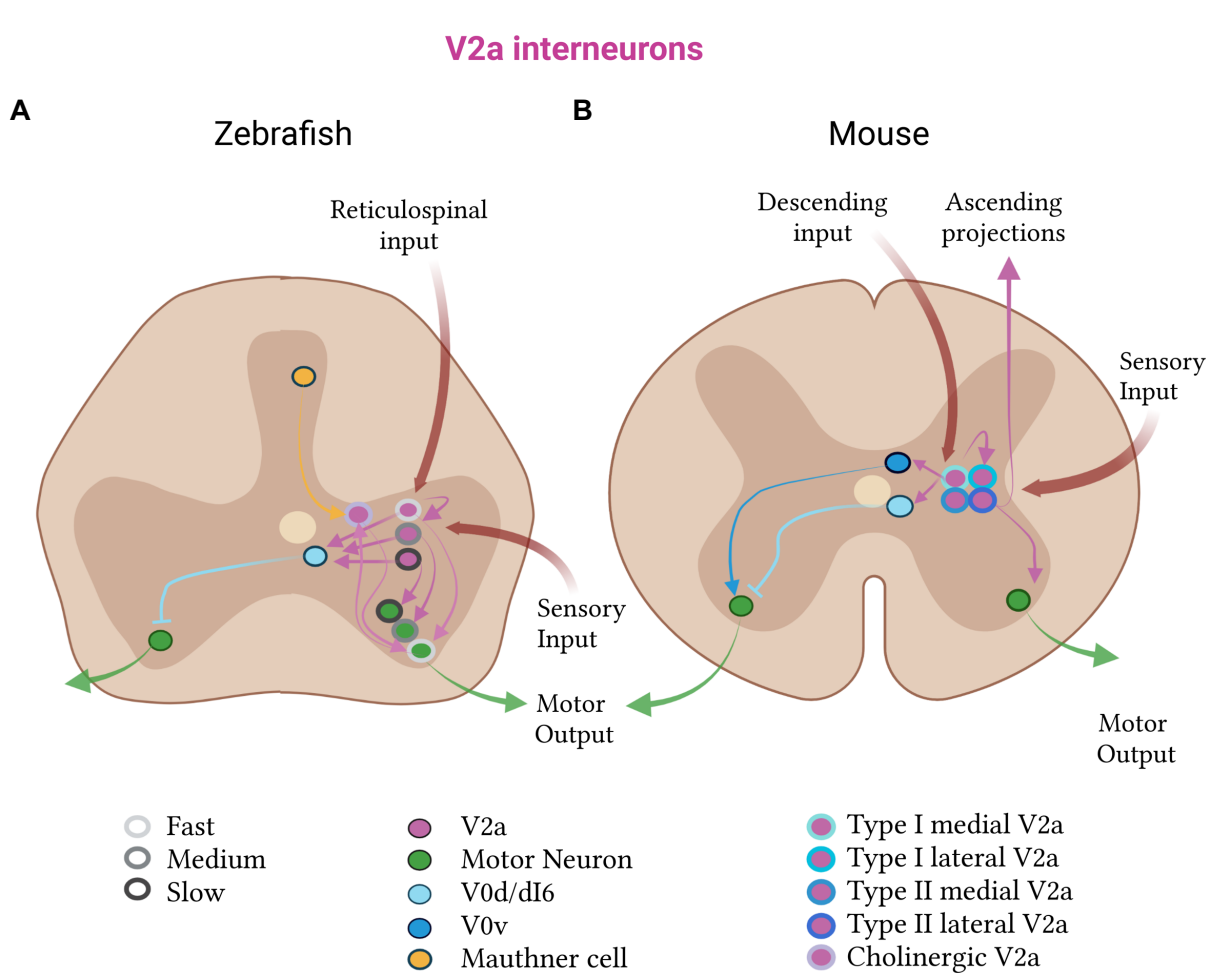
Excitatory V2a subtypes in zebrafish and mice. **(A)** In zebrafish, the V2a class (pink) is divided into fast (light gray, outline), medium (medium gray, outline) and slow (dark gray, outline) subtypes. The fast subtypes are more dorsal than the slow subtypes. Each subtype receives reticulospinal and sensory input, and projects to the corresponding fast, medium or slow class of motor (green) and V0d (light blue) neurons. Zebrafish also have a cholinergic subclass of V2a neurons, which receive input from Mauthner cells and have bidirectional connections to fast motor neurons. **(B)** In mice, the V2a population receives descending and sensory input, projects to inhibitory V0d and motor neurons, and has recurrent connections. Mouse V2a neurons subdivide into type I and type II, which further divide into medial and lateral subtypes (shades of blue, outlines). Type I V2a neurons connect to V0 neurons (V0d in light blue and V0v in dark blue); type II V2a neurons have ascending connections.

The physiological role of V2a neurons in the activation and speed-dependent modulation of motor output was shown to map onto three spatially distinct microcircuits: one for driving slow, one for intermediate and one for fast motor neurons (Figure 2A; Kimura et al., 2006; Ampatzis et al., 2014). These circuit modules are spatially segregated along the dorsoventral axis of the spinal cord and arrayed such that the slow is recruited before the fast one (Song et al., 2018). Each module preferentially targets either slow or fast motor neurons and has different anatomical and functional properties (Song et al., 2018). The slow V2a neurons preferentially target slow motor neurons, have unidirectional caudally-projecting axons, and exhibit bursting activity. They display significant short-term potentiation, which decreases the number necessary to activate motor neurons. The fast V2a neurons preferentially target fast motor neurons, show no bursting activity, and project in both the caudal and rostral directions. They lack short-term potentiation and produce a weaker excitatory drive, requiring a larger number of them to be activated to generate a motor neuron response. This precise connectivity ensures that V2a excitatory neurons from one speed class induce strong EPSPs in motor neurons of the same, but not the other, speed class. The excitatory drive to V2a neurons and motor neurons of the same speed class is further organized in a continuum, such that at faster locomotor speeds, the drive to the intermediate and fast class is increased (Ampatzis et al., 2014). This circuit organization allows for a smooth transition from slow to fast swimming with increased drive.

The functional role of V2a speed-dependent microcircuit segregation is also evident during zebrafish development. Paralleling the development of swim behavior, in which strong contractions are needed for escape swimming at early stages while slow, sustained swimming only emerges at late stages, the dorsal, fast V2a class is formed earlier than the ventral, slow class (Kimura et al., 2006). In line with this finding, V2a neurons in early-stage larvae display two morphologically distinct classes: those with ascending and descending axons, and those with only descending axons (Menelaou et al., 2014). Within the descending population, the more dorsal V2a neurons project axons for longer distances and have higher synapse densities onto proximal motor neurons than the ventral ones (Menelaou et al., 2014). Such an organization is suitable for activation of the entire motor pool by the dorsal V2a population during fast escape swimming. The more ventral V2a population, in contrast, innervates smaller motor neuron territories and is active in-phase with motor neurons, thus providing finer motor control for late-stage slow swimming (Menelaou and McLean, 2012). This again supports the idea of dorsal V2a neurons being active in fast escape and the more ventral class being active during slow, sustained swimming.

An alternative V2a subdivision has recently been proposed in which molecular, morphological and electrophysiological, instead of anatomical and speed-dependent, features segregate these neurons into two groups (Menelaou and McLean, 2019). Two classes were proposed to control either timing or amplitude. V2a “timing” neurons preferentially connected to other V2a excitatory neurons and V0d inhibitory neurons. V2a “amplitude” neurons, in contrast, predominantly contacted motor neurons. An organization was proposed in which “timing” V2a neurons with dense electric interconnections receive inputs from primary afferents and set the frequency of locomotion. This group then relays the drive via strong glutamatergic excitation to the “amplitude” group of V2a neurons which controls the strength of movement, and to V0d inhibitory neurons which controls left–right coordination (Menelaou and McLean, 2019). This alternative subdivision of the V2a class is compatible with the prior one but captures a different aspect of circuit function.

Recent studies have found that zebrafish V2a neurons also include a cholinergic subpopulation which forms an integral part of the escape swim circuit (Guan et al., 2021). These neurons form bidirectional, electrical connections with ipsilateral motor neurons. Their ablation impairs the escape swim response, preventing the amplification and distribution of the escape command. Future experiments will determine how this cholinergic subpopulation relates to the other subpopulations established previously.

It is therefore clear that anatomically- and physiologically- distinct subclasses of ipsilateral V2a excitatory neurons exist in the zebrafish. This contrasts with the lamprey and larval *Xenopus* tadpole, where there is little evidence for such defined subclasses (Buchanan and Grillner, 1987; Zelenin et al., 2001; Soffe et al., 2009; Picton et al., 2018). These subdivisions are likely responsible for producing smooth muscle activation, as well as setting the strength and frequency of the varied swimming patterns of developing zebrafish. Future studies will reveal whether molecular differences underlie these anatomical and physiological distinctions.

### Mouse V2a neurons

As in zebrafish, V2a excitatory neurons in mouse provide excitation to motor neurons and regulate speed-dependent microcircuits. This conserved function in driving locomotion and regulating its tempo is supported by experiments both *in vitro* and *in vivo* (Crone et al., 2008). In isolated spinal cord preparations, the absence of V2a neurons affects locomotor burst amplitude and cycle period (Crone et al., 2009; Zhong et al., 2011). In mice which lack these neurons, an abnormal transition from an alternating trot-like to a synchronized galloping gait occurs at high speeds (Crone et al., 2009). Ablating this class also leads to a partial uncoupling of the two sides of the spinal cord in mice (Crone et al., 2008). Using anatomical tracing, it was demonstrated that V2a excitatory neurons project to commissural interneurons, including a set of molecularly defined V0 neurons which produce left–right alternation (Crone et al., 2008). Together, these experiments demonstrate a conserved role for V2a neurons in the drive and maintenance of a stable rhythm, and left–right coordination, across vertebrates.

Given these varied functional properties, it is not surprising that multiple V2a subtypes have been identified in mice. When stimulated, individual V2a excitatory neurons showed one of four electrophysiological responses (Zhong et al., 2011). They either (i) fired rhythmically in-phase with ventral root activity, (ii) had subthreshold rhythmic activity and lower input resistance, (iii) showed non-rhythmic tonic firing, or (iv) were silent. The latter two groups did not increase their firing frequency or recruitment with increased frequency of locomotion, while the former two groups did. This is consistent with the finding that half of V2a neurons receive rhythmic locomotor synaptic drive, while the other half do not (Zhong et al., 2011). The locations of the neurons within the spinal cord could not predict their firing properties (Kwan et al., 2010; Zhong et al., 2011).

It is now becoming clear that supraspinal communication is crucial in defining these physiologically-distinct V2a subtypes. Hayashi and colleagues recently split the V2a population by marker expression and projection pattern (Hayashi et al., 2018). Type I V2a excitatory neurons have high VSX2 expression and form recurrent connections with neighboring spinal neurons, constituting the local V2a subpopulation. Type II V2a excitatory neurons downregulate VSX2 at later stages of development and have ascending projections, constituting the non-local V2a subpopulation. The two subtypes were found to form counter-gradients with each other, with type I cells forming the majority of the lumbar V2a population and type II, the majority at cervical levels.

Single-cell RNA sequencing demonstrated that these two groups could further be split into multiple molecularly defined subtypes, including a lateral and medial subpopulation. The lateral subpopulation of V2a neurons was selectively enriched at limb levels, while the medial subpopulation was evenly distributed along the rostro-caudal axis. This suggests that the medial subpopulation might represent a shared axial musculature network, while the lateral, might be responsible for differences in fore- and hindlimb dexterity (Hayashi et al., 2018), a feature unique to four-limbed vertebrates such as mice. A large proportion of the lateral, cervical population were type II V2a neurons, in agreement with the importance of supraspinal communication in the facilitation of fine control of the forelimbs. In direct support of this, selective ablation of cervical V2a neurons perturbed reaching while leaving other movements intact (Azim et al., 2014). The prevailing hypothesis is that these level-specific V2a cell-type specializations give rise to the vast differences in motor repertoire between the fore- and hindlimb.

The heterogeneity of the excitatory ipsilateral V2a class in mice aligns with the conserved property of graded muscle recruitment with increasing locomotor speed and the tetrapod-specific requirement to control limb movements. The V2a excitatory neurons in mice appear to differ more based on their position along the body axis than in zebrafish. This is consistent with the ability of mice to perform different motor behaviors at each body level.

### Cross-species perspective on V2a

Common and divergent properties of V2a neurons have emerged from these studies across species. In all vertebrates, the V2a population serves as an important source of excitatory drive for locomotion. Notably however, they do not appear to be necessary for rhythm-generation in mice. In higher order vertebrates such as zebrafish and mice, functional, anatomical and molecular V2a subdivisions seem to confer new physiological roles in movement pattern regulation. The first new property to emerge is the differential recruitment of V2a excitatory neurons at different speeds. This suggests that, contrary to the “size principle” which states that all motor neurons receive the same drive and are recruited depending on their size, locomotor drive is in fact specifically channeled to certain motor pools by V2a subpopulations (Ampatzis et al., 2014).

Functional distinctions between V2a neurons seem to be also reflected in their connectivity patterns. In lamprey and *Xenopus* tadpoles, V2a-like cells are connected to motor neurons, other V2a-like cells and commissural inhibitory interneurons (Buchanan and Grillner, 1987; Parker and Grillner, 2000; Li et al., 2006, 2009). These connections are also present in fish and mice, along with supraspinal projections (Kimura et al., 2006, 2013; Crone et al., 2008; Dougherty and Kiehn, 2010; Zhong et al., 2010; Ampatzis et al., 2014; Menelaou et al., 2014; Hayashi et al., 2018; Menelaou and McLean, 2019). In mice, V2a neurons are functionally heterogeneous (Schwenkgrub et al., 2020). Their subdivision into those that receive locomotor-related drive and those that do not (Zhong et al., 2011) may correspond to the two groups found in zebrafish. These groups were proposed to separately control timing and amplitude of locomotion (Menelaou and McLean, 2019), although further experiments are needed to validate this hypothesis.

In summary, V2a excitatory neurons have a vital role in driving movement. In zebrafish, this role has become specialized with their subdivision into strong and weak groups, differentially active in escape and slow swimming. In mice, it seems that only half of the V2a neurons receive locomotor-related drive and the function of the other half is unknown. The more cervical population may facilitate precise motor control of the forelimbs by relaying an efference copy of the motor command to supraspinal structures. Moreover in mice, V2a neurons have a speed-specific role in maintaining left–right alternation at high, but not low, frequencies, likely by exciting V0 interneurons. Given these varied roles, it is thus likely that the V2a population in mice is more molecularly diverse than in zebrafish. Single-cell sequencing can help us to address this hypothesis more directly in the future.

## V3 excitatory neurons

**Figure 3:**
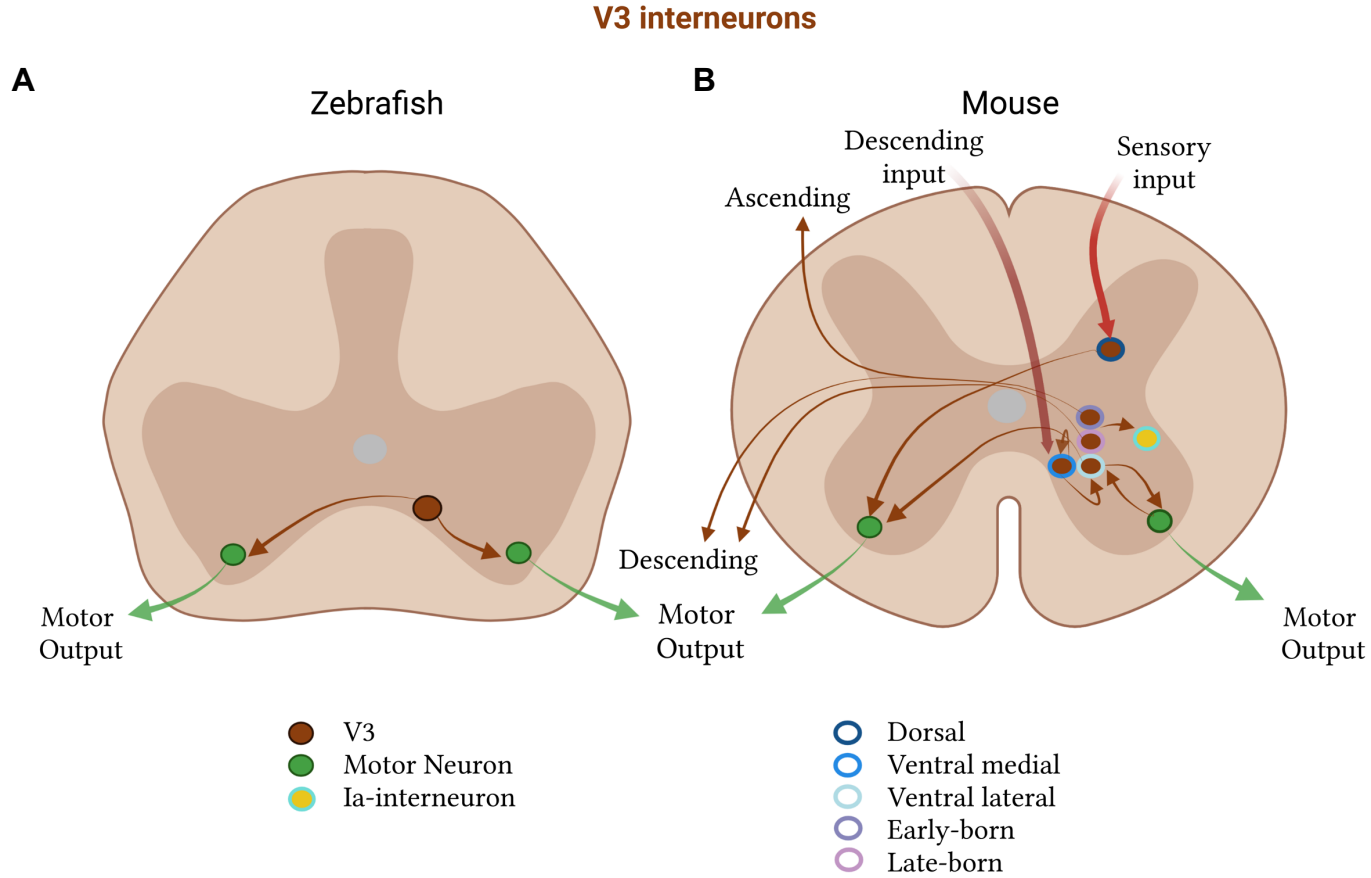
Excitatory V3 subtypes in zebrafish and mice. **(A)** In zebrafish, V3 neurons (brown) project to motor neurons (green) on both sides of the spinal cord and **(B)** in mice are divided into a dorsal (dark blue, outline) and ventral class, the latter of which is further subdivided into a medial (medium blue, outline) and lateral (light blue, outline) group. The ventral medial group receives descending commands, forms recurrent connections with itself, and projects to the ventral lateral group. The lateral group projects to motor neurons on the ipsi and contralateral side. Motor neurons project back to V3 neurons in mice. V3 neurons also subdivide by their birth time into an early-(dark purple, outline) and a late-born (light purple, outline) subpopulation, which form either both an descending and ascending, or only a descending, projection, respectively. V3 neurons in mice also project to Ia- interneurons.

V3 excitatory neurons, best studied in mice, are glutamatergic SIM1-expressing neurons that derive from the p3 progenitor domain during development (Briscoe and Ericson, 1999; Sugimori et al., 2007; Zhang et al., 2008; Yang et al., 2010). Their anatomical projection pattern serves as one of their best defining features. Like V2a neurons, they project ipsilaterally but, unlike them, the majority of V3 neurons also have contralateral projections that extend caudally (Figure 3; Zhang et al., 2008). Such bilaterally-projecting neurons do not seem to be part of the pattern-generating network in the lamprey spinal cord (Buchanan, 1982, 1999; Buchanan and McPherson, 1995; Cangiano and Grillner, 2005; Kozlov et al., 2009). They are however present in zebrafish, where they occupy a single ventromedial domain across the length of the spinal cord and match the projection properties of the anatomically-defined ventral medial (VeMe) neurons (Figure 3A; Hale et al., 2001; Higashijima et al., 2004a,c; Wiggin et al., 2022). In contrast, in the mouse, the V3 neurons migrate after differentiation to form spatially and physiologically distinct subpopulations (Figure 3B; Borowska et al., 2013). This poses the question of whether V3 subdivision is important for more complex locomotion in limbed, as opposed to finned, vertebrates.

### Zebrafish V3 neurons

Zebrafish V3 excitatory neurons comprise a spatially and functionally homogenous population of neurons active during fictive swimming (Figure 3A; Böhm et al., 2022; Wiggin et al., 2022). They are critical for the recruitment of motor neurons, as their activation increases swim strength and ablation reduces motor neuron activity (Böhm et al., 2022; Wiggin et al., 2022). They regulate the burst amplitude independent of the burst frequency, thus providing drive but not influencing the speed of swimming or underlying locomotor rhythm (Böhm et al., 2022; Wiggin et al., 2022). One proposed function of the zebrafish V3 population is the relay of excitatory drive to coordinate motor units, according to the desired amplitude of locomotor bursts. The bilateral and descending projection pattern of the V3 class in zebrafish is consistent with this hypothesis, as they contact multiple motor units. Moreover, the timing of V3 spikes relative to those of motor neurons, suggest a function in providing excitatory drive during locomotion (Böhm et al., 2022; Wiggin et al., 2022).

### Mouse V3 neurons

In mice, V3 neurons play a more specific role in the symmetry of motor control by ensuring the balance of motor output between the left and right sides of the spinal cord (Zhang et al., 2008). Suppression of V3 synaptic transmission in mice leads to loss of coordination and stability during locomotion, increasing the coefficient of variation, but not the mean, of both the duration and period of locomotor bursts (Zhang et al., 2008). Activation of V3 neurons increases the burst duration of motor neurons predominantly on the contralateral side, resulting in slowed locomotion (Danner et al., 2019). This suggests that the function of V3 excitatory neurons diverged during evolution to take on different roles in axial and tetrapod movement, as the latter requires more precise and fine-tuned balance between both sides of the spinal cord.

V3 neurons in mice are highly spatially and functionally heterogeneous, a property not observed in zebrafish (Figure 3B). These interneurons are distributed along the dorsoventral and rostrocaudal axes in the postnatal spinal cord, and are found in multiple laminae (Zhang et al., 2008; Borowska et al., 2013; Blacklaws et al., 2015). They can additionally be split into dorsal and ventral subpopulations, each with distinct electrophysiological and morphological properties (Borowska et al., 2013). Accordingly, dorsal V3s are proposed to relay sensory information as they are only active during running, while ventral V3s are active during both swimming and running (Borowska et al., 2013). Moreover, the dorsal group exhibits a complex branching morphology, low gain and diverse firing properties. The ventral group has a simple morphology, high gain, and constant tonic firing, making them more suited to faithfully relay and distribute motor commands.

The ventral V3 group can be further subdivided into a lateral and medial population in mice (Chopek et al., 2018). The lateral population excites both contra- and ipsilateral motor neurons, making bidirectional connections with ipsilateral motor neurons to provide them with glutamatergic recurrent excitation. Their contralateral targets are unidentified (Chopek et al., 2018), but are likely to also be motor neurons, as many V3-derived synapses can be found on motor neurons on the contralateral side (Zhang et al., 2008). Medial V3 neurons occupy a separate layer within the ventromedial spinal cord (Chopek et al., 2018), a layer which receives direct descending motor commands to initiate movement (Matsuyama et al., 1988). They form synapses with other medial and lateral ventral V3 excitatory neurons (Chopek et al., 2018). Therefore, it seems that the medial V3 excitatory neurons may integrate descending reticulospinal input, which they could then pass onto the lateral population to distribute the drive to the appropriate motor pools.

Recent work has also revealed that V3 neurons can be subdivided based on birth date and projection pattern (Deska-Gauthier et al., 2020; Zhang et al., 2022). The early-born V3 neurons have both ascending and descending commissural projections, while the late-born neurons have descending and local commissural projections. These two groups are also separated by location, with the early-born forming subgroups across the dorsoventral spinal laminae, and the late-born limited to ventral laminae (Deska-Gauthier et al., 2020). It was found that at least some ascending projections from lumbar V3 neurons terminate in contralateral cervical areas. These ascending V3 neurons are thought to be crucial for diagonal limb synchronization and gait coordination. Modeling has supported their importance in diagonal inter-limb coordination during trot in mice (Zhang et al., 2022). In the model, removing just the ascending V3 neurons eliminates trot, while maintaining gallop and bound, generating a powerful prediction to test with future experiments (Zhang et al., 2022). This selectivity of V3 subpopulations for a specific gait again highlights the additional specialization of spinal neurons for limbed movement in mice, as compared to fish.

### Cross-species perspective on V3

V3 excitatory neurons are similar in fish and mice, but more complex in the latter. This complexity arises from the division of the population into spatially distinct subpopulations, each specialized for a particular function. The split into dorsal and ventral subpopulations is likely to be important for integrating sensory information in limbed vertebrates. The further division of the ventral population into medial/lateral and early/late-born subgroups may allow for more fine-grained control over limb muscles, by providing a mechanism for distributing drive to specific motor pools on the ipsi- and contralateral side.

In addition, it was previously shown in mice that stimulation of motor neurons can initiate locomotor-like activity (Mentis et al., 2005; Chopek et al., 2018) and influence locomotor rhythm via a glutamatergic pathway (Falgairolle et al., 2017; Chopek et al., 2018). The bidirectional connections of ventrolateral V3 excitatory neurons with motor neurons, a property described in mice, may therefore be important for allowing motor neurons to contribute to the locomotor rhythm and maintain the balance of activity between both sides of the spinal cord. Additionally, V3 neurons in mice were shown to synapse on inhibitory Ia-interneurons, which could facilitate burst termination and provide another layer of control over the locomotor rhythm (Zhang et al., 2008). These findings are also in accordance with the proposed roles of V3 neurons in facilitating gait transitions in mammals (Rybak et al., 2006; Danner et al., 2017; Zhang et al., 2022).

Across species, the V3, like the V2a, population contributes to excitatory drive. In zebrafish they seem to be important for the recruitment of motor units and regulation of motor burst amplitude. In mice, they maintain the balance of activity on both sides of the spinal cord, and integrate, relay and direct sensory information and descending motor commands. Molecular studies will be important for understanding the relationship between gene expression and these divergent functions. Toward this end, recent studies have shown that V3 neurons in mice and zebrafish are molecularly heterogeneous (Mukaigasa et al., 2021). This raises the possibility that these molecular subdivisions may, in mice, delineate the known anatomical and functional differences between subpopulations and, in zebrafish, define diverse, as of yet unknown, subpopulations for movement pattern generation.

## V0 excitatory and inhibitory commissural neurons

Commissural interneurons are a common feature of all vertebrate spinal cords. In the lamprey, both excitatory glutamatergic and inhibitory glycinergic commissural interneurons have been identified (Ryczko et al., 2010). They can be divided into three groups based on their reticulospinal inputs (Buchanan, 1982, 1999; Shupliakov et al., 1992; Buchanan and McPherson, 1995; Mentel et al., 2006, 2008; Biró et al., 2008). Excitatory commissural neurons in lamprey seem to be selectively involved in the movement of the dorsal fin (Shupliakov et al., 1992; Mentel et al., 2006, 2008). They are active in phase with both ipsi- and contralateral fin motor neurons, and drive their simultaneous activation during straight swimming. Inhibitory commissural neurons have been shown by modeling and experimental studies to be necessary for the generation of bilateral undulatory swimming, but not unilateral rhythm generation (Cohen and Harris-Warrick, 1984; Alford and Williams, 1989; Hagevik and McClellan, 1994; Aoki et al., 2001; Cangiano and Grillner, 2003, 2005; Huss, 2007; Kozlov et al., 2009). They receive input from excitatory ipsilateral neurons, and, on the contralateral side, drive motor neurons, lateral inhibitory neurons, and other inhibitory commissural neurons (Buchanan, 1982, 1999). They decrease burst frequency and coordinate and stabilize the activity on both sides of the spinal cord (Buchanan, 1999; Cangiano and Grillner, 2005; Kozlov et al., 2009).

In other vertebrates such as zebrafish and mice, ventral commissural interneurons, termed V0 neurons, derive from the p0 progenitor domain and are characterized by expression of the transcription factor DBX1, which is required for their development and commissural connectivity (Moran-Rivard et al., 2001; Pierani et al., 2001; Kiehn, 2006). In both zebrafish and mice, they extend axons rostrally for two to four spinal cord segments, and either mono- or poly-synaptically synapse onto motor neurons, suggesting that they are important for diagonal coordination (Moran-Rivard et al., 2001; Pierani et al., 2001; Butt and Kiehn, 2003; Lanuza et al., 2004; Quinlan and Kiehn, 2007; Svara et al., 2018; Ronzano et al., 2021). In zebrafish, there is evidence that the V0d population forms reciprocal connections with itself, and projects to ipsilateral inhibitory neurons (Soffe et al., 1984; Satou et al., 2020). Additionally, both V0d and V0v populations likely connect to V2a neurons and motor neurons (McLean et al., 2008; Griener et al., 2015; Svara et al., 2018; Satou et al., 2020; Roussel et al., 2021). In mice and rats, the V0 class is also thought to form reciprocal connections with other commissural neurons, and project to ipsilateral inhibitory neurons on the opposite side of the spinal cord (Kjaerulff and Kiehn, 1997; Birinyi et al., 2003; Butt and Kiehn, 2003; Quinlan and Kiehn, 2007). Compared to the monosynaptic connections in zebrafish, the projections to motor neurons in mice tend to be disynaptic (Kjaerulff and Kiehn, 1997; Butt and Kiehn, 2003; Quinlan and Kiehn, 2007; McLean et al., 2008; Svara et al., 2018).

**Figure 4:**
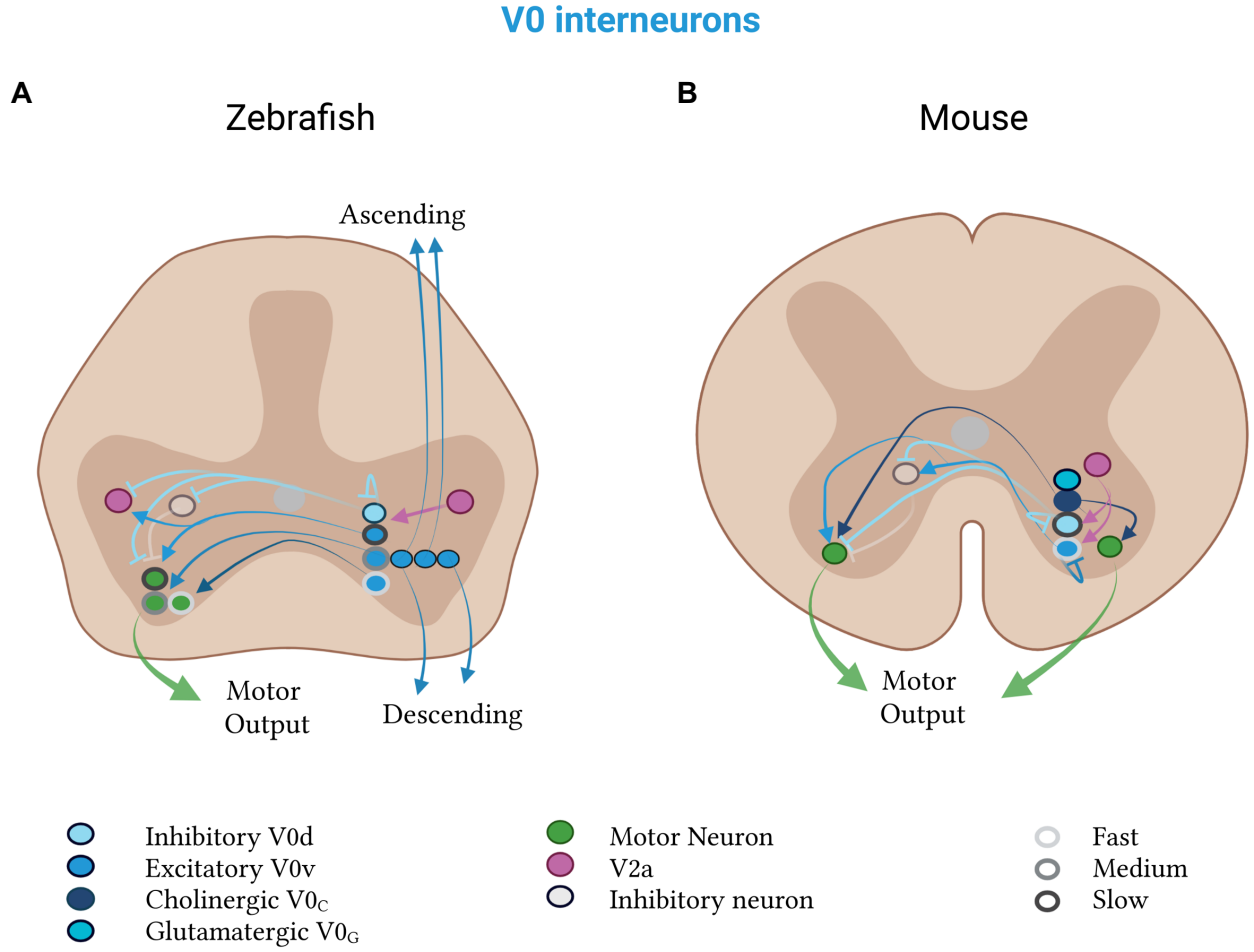
Mixed V0 subtypes in zebrafish and mice. V0d neurons (light blue) inhibit, and V0v neurons (medium blue) excite, contralateral motor neurons (green). **(A)** Zebrafish V0d neurons receive input from V2a neurons (pink) and project to other V0d, contralateral inhibitory (gray) and ipsilateral excitatory (pink) neurons. The V0v neurons project to V2a neurons and divide into a rhythmic and a non-rhythmic group. The rhythmic group is further split into fast (light gray, outline), medium (medium gray, outline) and slow (dark gray, outline) subtypes, which project to motor neurons of the respective speed class. Excitatory V0v neurons also segregate by projection pattern into ascending, descending and bifurcating subpopulations. **(B)** Mouse V0d and V0v neurons control slow and fast speeds, respectively. Both classes receive input from V2a neurons. The V0v class additionally projects to contralateral neurons (gray), which inhibit motor neurons. Mouse-specific V0c neurons (dark blue) are cholinergic and project to motor neurons on both sides of the spinal cord. Mouse-specific V0g neurons (turquoise) are glutamatergic and their projection pattern is as of yet unknown.

In zebrafish and mice, this V0 class is composed of both excitatory V0v and inhibitory V0d subtypes (Figure 4). Excitatory V0v interneurons derive from the ventral DBX1 progenitor domain and transiently express the homeodomain protein EVX1. Inhibitory V0d neurons derive from the dorsal DBX1 domain and lack EVX1 expression, but unlike V0v, express PAX7 (Moran-Rivard et al., 2001; Pierani et al., 2001). The V0d neurons are largely GABAergic or glycinergic, whereas the V0v neurons are glutamatergic (Moran-Rivard et al., 2001; Talpalar et al., 2013). As in the mouse and in contrast to the lamprey, extensive studies in zebrafish support that the V0 population is highly heterogeneous in its connectivity and function, with both the anatomically defined bifurcating multipolar commissural descending (MCoD) and unipolar commissural descending (UCoD) neurons best corresponding to the V0v class and the glycinergic commissural bifurcating longitudinal (CoBL) neurons, to the V0d class (Satou et al., 2012).

### Zebrafish V0 neurons

Zebrafish V0v and V0d neurons coordinate, regulate and drive a variety of locomotor features during fish development, highlighting their diverse contributions to higher order vertebrate movement. As with other interneuron classes in the zebrafish, they have historically been defined by their projection pattern, with the best studied being the multipolar commissural descending (MCoD) V0v subpopulation that develops at a later stage of neurogenesis (McLean and Fetcho, 2009; Satou et al., 2012). In late-stage larvae, V0v neurons are important for slow swimming (Ritter et al., 2001; McLean et al., 2007, 2008; McLean and Fetcho, 2009; Fidelin et al., 2015). They keep the head stable during this form of locomotion by coordinating the activities of diagonal trunk muscles, and thus enabling the fish to execute their characteristic S-shaped bends (Kawano et al., 2022). During fast swimming, V0v neurons are not active, and the head is no longer stable at these higher frequencies (McLean et al., 2008; McLean and Fetcho, 2009; Severi et al., 2014). Additionally, ablation of the V0v population decreased spontaneous swimming, suggesting that these neurons may also contribute to the general excitability of the motor circuit (McLean et al., 2008; Kawano et al., 2022). As zebrafish larvae mature into adults, the physiological properties of this class change. Adult V0v neurons display speed-dependent recruitment during swimming, with a large proportion recruited at fast speeds (Björnfors and El Manira, 2016).

The anatomical and electrophysiological properties of these V0v neurons are consistent with their role in coordinating diagonal activity during swimming (Figure 4A). Their axons cross the midline and descend, making direct monosynaptic connections with motor neurons that are contralateral and caudal to the presynaptic V0v neurons (Hale et al., 2001; McLean et al., 2008). They fire in a highly phasic manner, with their spike timing slightly preceding nearby motor neurons (Kawano et al., 2022). Modeling suggests that they may also connect to excitatory V2a neurons at later developmental stages (Roussel et al., 2021).

Reflecting these diverse physiological roles, V0v neurons in zebrafish are highly heterogenous. They can be divided into three subclasses based on temporal order of their development and their axonal projection patterns (Satou et al., 2012). The first to develop is the V0v subclass with ascending, then those with bifurcating, and finally, descending projections—with the ascending/descending corresponding to the unipolar UCoD and the bifurcating to the multipolar MCoD anatomical subclasses (Satou et al., 2012). Each of these subclasses is composed of rhythmic and non-rhythmic types. The rhythmic group can be further split into subsets which are recruited at slow, intermediate, or fast speeds (Satou et al., 2012), explaining the speed-dependent recruitment seen in adults.

Less is known about the inhibitory V0d neurons in zebrafish. Anatomically, the morphology of the glycinergic commissural bifurcating longitudinal (CoBL) population matches that of the V0d population (Satou et al., 2012). Like the inhibitory commissural interneurons in the lamprey, they likely provide mid-cycle inhibition onto motor neurons and other neurons, important for left–right motor activity (Satou et al., 2020). Both V0d and the Dmrt3a-expressing dI6 neurons (see below) have monosynaptic inhibitory connections to neuronal populations active during fictive swimming, including contralateral motor neurons (Figure 4A; Satou et al., 2020). The V0d population tends to fire during faster and stronger movements; while the dI6 subpopulation fires during normal fictive swimming (Satou et al., 2020). Both populations are active in phase with nearby motor neurons, suggesting that they inhibit motor neurons on the contralateral side of the spinal cord when the ipsilateral side is active—a property that is a crucial feature of undulatory swimming and conserved in the lamprey.

### Mouse V0 neurons

Like zebrafish, mice also have an excitatory V0v and an inhibitory V0d population (Figure 4B). Excitatory V0v neurons in mice coordinate diagonal limb muscles during walking, analogous to their role in diagonal muscle coordination during zebrafish swimming. Ablation of the V0v, or V2a neurons that innervate them, at the cervical level mainly impacts left–right hindlimb, but not forelimb or interlimb, coordination (Ruder et al., 2016). This suggests an evolutionarily conservation of this long-range, cross-body diagonal function of V0 neurons across species. In addition, mice which lack V0 neurons exhibit increased co-bursting between the left and right sides of both flexor and extensor muscles, which is expressed as quadrupedal hopping at all frequencies of locomotion (Lanuza et al., 2004; Talpalar et al., 2013). This further demonstrates that V0 neurons also contribute to left–right alternation in mice.

The conservation of V0 subtype function between species is also demonstrated by the similar role of zebrafish and mouse V0 neurons in speed-dependent motor control. Selective ablation of just the inhibitory V0d neurons leads to a lack of left–right alternation at slow locomotor speeds, mixed coordination at medium, and normal alternation at high speeds (Talpalar et al., 2013). Conversely, ablation of only V0v neurons has the opposite effect: normal alternation at slow speeds and hopping at intermediate and high speeds (Talpalar et al., 2013). This high-speed hopping is also observed in V2a mutants, as demonstrated by Crone et al. (2008). V2a excitatory neurons in mice project to V0v neurons, providing a circuit-level mechanism for this phenotype (Crone et al., 2008). Computational modeling suggests that V0v neurons may also project to contralateral inhibitory interneurons that contact motor neurons (Shevtsova et al., 2016; Danner et al., 2017). Thus, as in zebrafish in which V0 subpopulations are segregated by the speed of locomotion they influence; in mice, V0d control slow, and V0v high, speed locomotion.

Unlike zebrafish, there is emerging evidence in mice of two other excitatory classes of V0 neurons: the cholinergic V0_C_ and glutamatergic V0_G_ neurons (Zagoraiou et al., 2009). Both populations are marked by the transcription factor PITX2. V0_C_ neurons, the best studied of these two populations, provide cholinergic C-bouton input to motor neurons and V1-derived Ia interneurons on either one or both sides of the spinal cord (Figure 4B; Zagoraiou et al., 2009; Siembab et al., 2010; Stepien et al., 2010). Genetic inactivation of V0c output results in behavioral deficits in task-dependent motor performance (Zagoraiou et al., 2009). Their firing activity is tightly phase-locked to that of motor neurons. When they are inactivated, motor neuron firing and muscle activation are impaired (Zagoraiou et al., 2009). Their activation conversely increases the excitability of motor neurons by reducing hyperpolarization after each action potential (Miles et al., 2007). This suggests that V0c excitatory neurons activate motor neurons to ensure firing at rates appropriate for the desired locomotor task, a property of the V0 population that has thus far only been found in mice.

### Cross-species perspective on V0

Across vertebrates, the V0 class is important for long-range coordination of rostrocaudal and left–right body parts. One might therefore predict that these are the neurons that vary the most between tetrapods that differ in their default mode of locomotion along these axes, such as frogs and mice. Within the V0 class however, there is remarkable conservation of subtypes between species with excitatory/inhibitory, diagonal-coordinating and speed-dependent classes highly conserved. However, the V0_C_ and V0_G_ subclasses in mice, not present in zebrafish, are a notable exception to this conservation. Additionally, the V0v speed-dependent subclasses of zebrafish have not been investigated in mice, leaving open the question of whether this subdivision is conserved. Thus, V0 specialization quantitatively may not increase over vertebrate evolution but qualitatively may change due to each species’ unique left–right coordination requirements. It will be of interest to test whether these functional differences are also present at a molecular and anatomical level, and the extent to which they are conserved in simpler vertebrate species.

## V1 and V2b inhibitory ipsilateral neurons

In addition to commissural excitation and inhibition, ipsilateral inhibition is a key component of more complex swim and limb spinal circuits. Two types of inhibitory ipsilaterally projecting neurons exist in the lamprey: ipsilateral inhibitory neurons (IINs) and lateral inhibitory neurons (LINs; Rovainen, 1974; Buchanan and Grillner, 1987; Buchanan and Grillner, 1988). The IINs inhibit both motor and commissural neurons, while the LINs generally inhibit only commissural neurons (Buchanan, 1982; Buchanan and Grillner, 1988; Rovainen, 2011). LINS additionally receive inputs from ipsilateral excitatory, contralateral and dorsal neurons (Rovainen, 1974; Buchanan, 1982; Buchanan and Grillner, 1987). Originally, models of spinal rhythm-generating networks in lamprey incorporated ipsilateral inhibitory neurons (Wallen et al., 1992). However, based on the finding that ipsilateral inhibition is not required for the generation of a hemi-cord burst pattern (Cangiano and Grillner, 2003), these neurons are often no longer included and may instead be important for inhibiting dorsal fin motor neurons (Kozlov et al., 2007; Mentel et al., 2008; Kozlov et al., 2009). This led to the hypothesis that the dorsal fin circuit was the precursor to the lateral fin and, subsequently, the flexor-extensor limb circuit. In larval Xenopus swim circuits however, ipsilateral inhibitory neurons, termed aINs, are believed to be a key component of the tadpole’s pattern-generating network for escape swimming (Li et al., 2002, 2004a), implicating them in larval forms of undulatory swimming.

**Figure 5:**
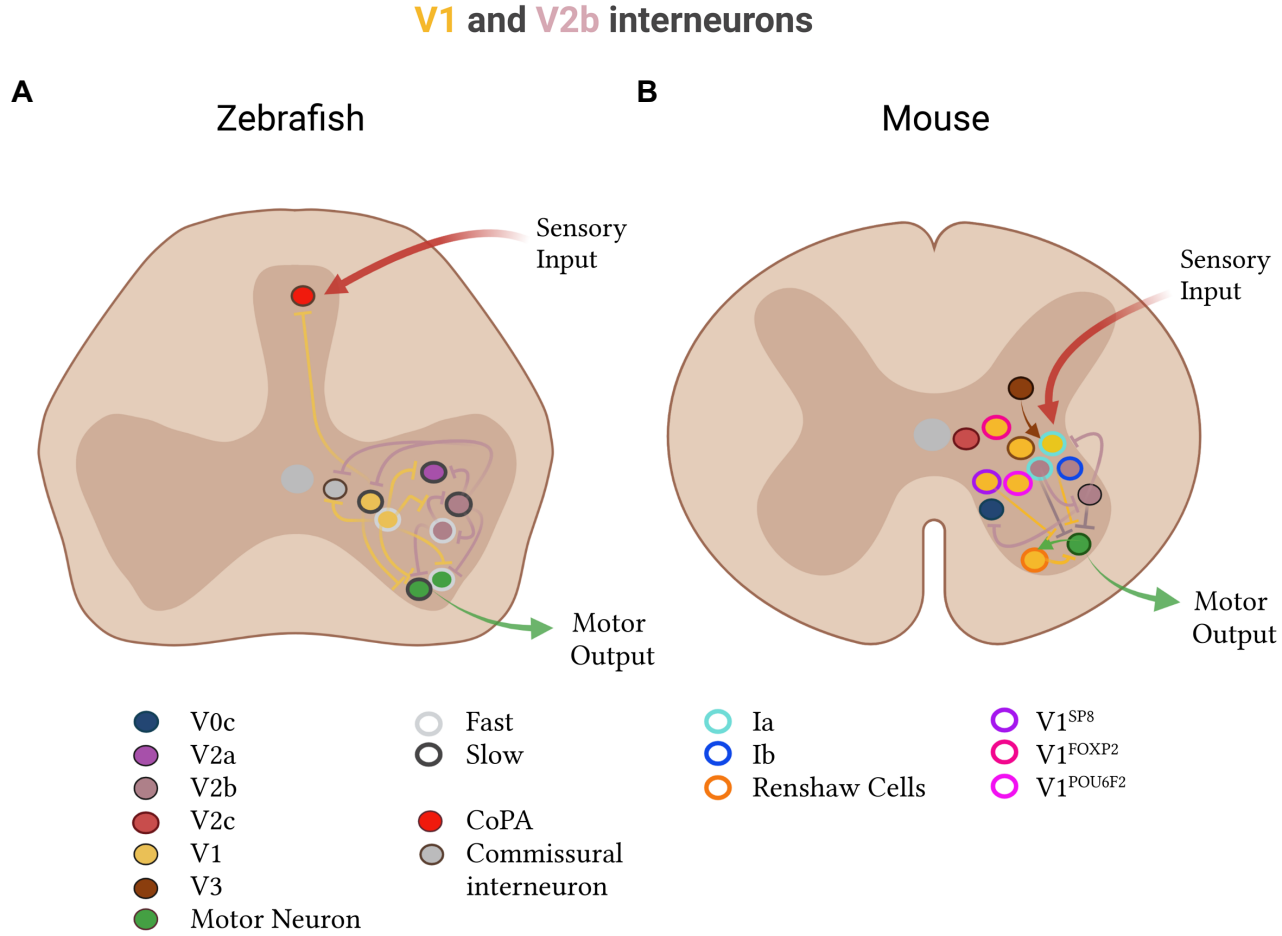
Inhibitory V1 and V2b subtypes in zebrafish and mice. **(A)** In zebrafish, V1 (yellow) and V2b (brown) divide into fast (light gray, outline) and slow (dark gray, outline) subtypes. V1 neurons: The fast V1 subgroup (yellow, light gray outline) inhibits both slow (green, dark gray outline) and fast (green, light gray outline) motor neurons in addition to slow-type V2a neurons (pink, dark gray outline). The slow V1 subgroup (yellow, dark gray outline) inhibits slow motor neurons (green, dark gray outline). V1 neurons also project to dorsal CoPA neurons (red) which receive sensory input, V2a neurons (pink), V2b neurons (brown), and commissural neurons (gray). V2b neurons: Slow V2b neurons (brown, dark gray outline) inhibit fast motor and other V2b neurons. Fast V2b neurons (brown, light gray outline) inhibit slow motor and other V2b neurons. V2b neurons in zebrafish also project to V2a, V1 and commissural neurons. **(B)** In mice, V1 neurons and subdivide into Ia-interneurons (light blue, outline), which receive sensory input and inhibit motor output; Renshaw cells (orange outline) which form recurrent connections with motor neurons; and four clades: Sp8 (purple outline), FoxP2 (pink-red outline) and Pou6f2 (pink outline). V1 neurons also receive input from V3 neurons (brown). V2b neurons include Ia- and Ib- (dark blue, outline) interneurons. V2b-derived Ia-interneurons inhibit motor and other V2b neurons. V2b neurons also inhibit V0c neurons. An additional V2c class is present in mice (red-pink) with an unknown projection pattern.

In zebrafish and mice, ipsilateral inhibitory neurons fall into two types: the V1 and V2b inhibitory classes (Figure 5). V1 neurons derive from the p1 progenitor domain, express the transcription factor Engrailed-1 (EN1) and the neurotransmitters GABA and/or glycine, and target ipsilateral motor neurons and other ipsilateral inhibitory neurons (Saueressig et al., 1999; Higashijima et al., 2004b; Sapir et al., 2004; Alvarez et al., 2005; Lundfald et al., 2007; Batista et al., 2008; Betley et al., 2009; Siembab et al., 2010; Bhumbra et al., 2014; Zhang et al., 2014; Shevtsova and Rybak, 2016; Callahan et al., 2019; Kimura and Higashijima, 2019; Sengupta et al., 2021). In zebrafish, the homologs of the mammalian V1 neurons are the circumferential ascending (CiA) neurons, which similarly express En1, project ipsilaterally, and contact motor neurons, other ipsilateral inhibitory and excitatory neurons, and commissural neurons (Higashijima et al., 2004b; Li et al., 2004a; Kimura and Higashijima, 2019; Sengupta et al., 2021).

The V2b ipsilateral inhibitory population derives from the LHX3-expressing p2 progenitor domain in mice and is defined by the expression of the transcription factors GATA2/3 (Lundfald et al., 2007; Peng et al., 2007; Callahan et al., 2019). This GATA3-expressing population seems well-conserved across vertebrates and beyond, present in the spinal cord of chicks and even the ventral nerve cord of worms (Karunaratne et al., 2002; Vergara et al., 2017). In zebrafish, V2b inhibitory neurons are thought to be the anatomically defined ventral longitudinal (VeLD) neurons, which similarly express Gata3 and project ipsilaterally, but derive from the pMN domain (Bernhardt et al., 1992; Hale et al., 2001; Peng et al., 2007; Batista et al., 2008; Seredick et al., 2012). In both mice and fish, V2b neurotransmitter profiles change during development: a large proportion initially uses GABA (Batista et al., 2008), and later transitions to glycine (Lundfald et al., 2007; Zhang et al., 2014; Callahan et al., 2019). In mice, the p2 progenitor domain also gives rise to a third class of neurons, the V2c neurons, which express SOX1 and GATA3 transiently in early development (Panayi et al., 2010). The functional role and homology in zebrafish of this V2c population are not yet clear. Diversification of the V1 and V2b inhibitory populations however seems key for producing an expanded repertoire of movement patterns in mice, as compared to fish.

The V2b class largely projects their axons caudally in both zebrafish and mice (Lundfald et al., 2007; Britz et al., 2015; Callahan et al., 2019). In zebrafish, this class contacts motor neurons and many interneuron classes on the ipsilateral side of the spinal cord, including V2a, V1, V2b, and commissural neurons (Sengupta and Bagnall, 2022). In mice, there is evidence that they project to V0c neurons, V1 neurons, and motor neurons (Zhang et al., 2014; Shevtsova and Rybak, 2016). Notably, the well-studied Ia- and Ib- inhibitory interneuron populations, which control the basic flexion-extension and auto-inhibitory reflex circuits respectively, also derive from the V1 and/or V2b neuron types (Benito-Gonzalez and Alvarez, 2012; Britz et al., 2015). Together, V1 and V2b neurons are necessary for flexor-extensor alternation in mice (Zhang et al., 2014; Britz et al., 2015).

### Zebrafish and frog V1 and V2b neurons

The existence of the V1-homologous CiA and V2b-homologous VeLD neurons in zebrafish, which lack the same extent of flexor-extensor divisions as four-limbed vertebrates, suggests that flexor-extensor coordination was only a role that this population took on later in evolution or alternatively, with pectoral and pelvic fin control (Thorsen and Hale, 2007; Uemura et al., 2020). Ablation of V1 inhibitory neurons in both zebrafish and mice leads to reduced fictive locomotor speeds (Gosgnach et al., 2006; Kimura and Higashijima, 2019). This occurs via reduced inhibition and thus, a longer intersegmental delay and locomotor cycle period (Tegner et al., 1993; Tunstall and Roberts, 1994; Gabriel et al., 2008). In contrast, *in vivo* optogenetic suppression of V2b activity in zebrafish leads to an increase, and activation to a decrease, in tail beat frequency (Callahan et al., 2019). In the *Xenopus* tadpole, there is only one ipsilateral inhibitory neuron class, the ascending interneuron (aIN) population, which is thought to modulate the swim cycle by providing in-phase inhibition to motor and other rhythm-generating neurons (Li et al., 2004a; Roberts et al., 2008). Thus, in-cycle inhibition of locomotion represents a conserved feature of V1 neurons in motor pattern generation across vertebrates.

Along with their role in regulating the motor pattern, the V1 inhibitory class is also believed to play an essential part in sensory integration in both *Xenopus* and zebrafish, through reflex inhibition during movement via dorsal interneuron connectivity (Li et al., 2004a; Higashijima et al., 2004b; Knogler and Drapeau, 2014; Sengupta et al., 2021). This is reminiscent of the presynaptic inhibition of spinal sensory feedback necessary for smooth movement in mice. Although in mice, this function is likely carried out by dorsal interneurons (Fink et al., 2014).

Like motor and V2a excitatory neurons, V1 inhibitory neurons can be divided into slow and fast subtypes in zebrafish (Figure 5A; Kimura and Higashijima, 2019). The mechanism by which V1s regulate motor output in this speed-dependent manner is through direct connections with motor and V2a excitatory neurons (Kimura and Higashijima, 2019). During fast swimming, strong in-phase inhibitory inputs from fast-type V1 neurons suppress the activity of slow-type V2a and motor neurons (Kimura and Higashijima, 2019). During slow swimming, slow-type V1 inhibitory neurons act on slow swimming circuits, by providing inhibition to regulate the cycle frequency. When swimming changes from slow to fast, fast-type V1 neurons are thought to shut down the slow circuit. In parallel, the fast subpopulation regulates the cycle period of fast circuits, tuning their inhibition to the strength of excitation they receive (Kishore et al., 2014; Kimura and Higashijima, 2019). This regulation is important for deactivating slow muscles and slow-type motor neurons during fast swimming (Tsukamoto, 1984; Jayne and Lauder, 1994; Buss and Drapeau, 2002; Menelaou and McLean, 2012; Kishore et al., 2014).

V2b inhibitory neurons in zebrafish can be divided into two subclasses by neurotransmitter and morphological properties: V2b-mixed and V2b-gly subpopulations (Figure 5B; Callahan et al., 2019). Both express glycine, with the V2b-mixed also expressing GABA. The two subtypes are indistinguishable in their physiology and are found along the entire rostrocaudal axis, with the V2b-mixed more ventral and V2b-gly more dorsal in its position. Both classes synapse directly onto motor neurons but target speed-specific circuits: the V2b-mixed targets slow, and the V2b-gly targets fast, motor neurons. However, the V2b-gly subclass innervated more of the dorsal spinal cord than the V2b-mixed type (Callahan et al., 2019). Additionally, rostral V2b neurons inhibit more caudal V2b neurons, leading to circuit disinhibition at long-range. Locally, V2b-mixed also make reciprocal connections onto V2b-gly neurons, and vice versa, which may be important for stabilizing the circuit at a desired speed (Callahan et al., 2019).

### Mouse V1 and V2b neurons

Similar to zebrafish, ipsilateral inhibitory neurons in mice are also required for regulating locomotor speed. *Pax6-*knockout mice, which lack V1 inhibitory neurons, display prolonged motor neuron activation which leads to slowed stepping, a phenotype that is replicated when V1 inhibitory neurons are acutely silenced or hyperpolarized (Gosgnach et al., 2006; Falgairolle and O’Donovan, 2019). More recent studies in the mouse have revealed additional heterogeneity in V1 function, with the type of manipulation producing different effects on the frequency of motor output (Falgairolle and O’Donovan, 2019; Falgairolle and O’Donovan, 2021). This supports the further subdivision of V1 interneurons by connectivity into functionally distinct subpopulations, a phenomena previously proposed in computational models of V1 circuits (Shevtsova and Rybak, 2016).

Gain- and loss-of-function experiments in mice also show that V1 and V2b neurons contribute directly to the coordination of limb movement. Mice lacking V1 inhibitory neurons have defects in flexor-extensor alternation: during the step cycle, they exhibit defective extension and prolonged flexion, causing an overall hyperflexion of the limb (Britz et al., 2015). Mice lacking V2b inhibitory neurons show an increase in extension and a lack of flexion, causing an overall hyperextension of the limb (Britz et al., 2015). Optogenetic activation of V2b neurons also suppresses extensor activity (Britz et al., 2015). It is therefore believed that V1 neurons restrict flexor activity during stance and facilitate the swing-to-stance transition, while V2b neurons facilitate the stance-to-swing transition by suppressing extensor activity during swing (Britz et al., 2015). The Ia-interneurons, derived from V1 and V2b neurons, and Ib-interneurons, derived only from V2b neurons, are the predominant neuron types for controlling flexor-extensor alternation (Akay et al., 2014; Britz et al., 2015).

Blocking both V1 and V2b inhibitory neuron-derived neurotransmission in the isolated mouse spinal cord leads to synchronous flexor and extensor activity and marked deficits in limb-driven movements, but normal left–right alternation (Zhang et al., 2014). Conversely, the commissural interneurons that contribute to left–right alternation (see V0 section above) do not affect flexor-extensor alternation (Whelan et al., 2000; Kiehn, 2006; Zhang et al., 2014). Two conclusions can thus be made from these findings. First, the V1 and V2b neurons are exclusively responsible for controlling flexor-extensor alternation by acting on the ipsilateral spinal cord. Second, the rhythm-generating circuits on each side of the spinal cord are largely decoupled from the ones that control alternation across the cord.

One unique feature of V1 inhibitory neurons in mice is their well-characterized physiological and transcriptional subtype diversity (Bikoff et al., 2016). In mice, the V1 class includes the well-studied Renshaw cells and reciprocal Ia-interneuron subtypes which are believed to play a role in flexor-extensor inhibition (Eccles et al., 1956; Feldman and Orlovsky, 1975; Sapir et al., 2004; Alvarez et al., 2005; Benito-Gonzalez and Alvarez, 2012; Stam et al., 2012; Zhang et al., 2014). Notably, recurrent and reciprocal V1 types make up <25% of the V1 class (Sapir et al., 2004; Alvarez et al., 2005), leaving open the question of what constitutes the other 75% of neurons. More recent studies have shown that the V1 class can be grouped into around 50 distinct subtypes, or four clades, based on combinatorial expression of FOXP2, SP8, POU6F2 and other transcription factors (Bikoff et al., 2016; Gabitto et al., 2016; Sweeney et al., 2018). Each clade has a distinctive settling position, physiology and synaptic connectivity (Bikoff et al., 2016; Gabitto et al., 2016).

Settling position in particular constrains neuronal input specificity, forming inhibitory microcircuits that selectively act on the motor pools innervating each proximodistal muscle, exemplified by differences in V1 to MN connectivity for the hip, knee and ankle (Bikoff et al., 2016). Segmental differences in transcriptionally defined V1 subsets at limb- and non-limb levels of the spinal cord have also been observed (Francius et al., 2013; Sweeney et al., 2018). This vast transcriptional heterogeneity suggests a parallel amount of anatomical or functional diversity. One possibility is that it is necessary for motor pool innervation and coordination. If this were the case, similar heterogeneity would be expected in the V2b neurons – which has just begun to be examined at a molecular level (Francius et al., 2013). Nonetheless, high levels of molecular heterogeneity in the ipsilateral inhibitory neurons seems to be key for producing an expanded repertoire of movement patterns in mice, as compared to zebrafish.

### Cross-species perspective on V1/V2b

In aquatic vertebrates, V1 and V2b inhibitory neurons control the speed of swimming and ensure faithful rostral-caudal propagation of activity. In tadpoles and zebrafish for example, they provide in-phase inhibition to the CPG, including motor neurons, to regulate the length of each swim bout. In zebrafish specifically, V1 and V2b inhibitory neurons can be split into speed-specific subtypes and act as a brake on the locomotor circuitry.

It is likely that the zebrafish circuit organization is also present in the tadpole. Li et al. (2004a) demonstrated the presence of direct connections between aINs (corresponding to V1 inhibitory neurons) and dINs (likely V2a excitatory neurons). The aINs are known to provide early-phase inhibition to motor neurons. There was a strong correlation between aIN-derived inhibitory inputs and the frequency of swimming (Li et al., 2004a).

In addition to their shared role in the regulation of motor output across vertebrates, V1 and V2b neurons are specialized for flexor-extensor coordination in limbed vertebrates such as mice (Britz et al., 2015). Their innervation patterns are biased in their connectivity with flexor and extensor motor pools to ensure smooth transitions through the step cycle (Britz et al., 2015). Moreover, in tetrapods, this idea of motor pool specialization of ipsilateral inhibitory circuits can be extended further, as the settling position of V1 neurons predicts their subtype and innervation patterns (Bikoff et al., 2016). It is thus likely that the diversity of V1 neurons may also enable other aspects of motor pool coordination such as fine motor control, which remains to be tested and is a crucial difference between vertebrate species.

## Other ventral neurons

There are two additional types of ventral interneurons identified in mice, which have not been assigned to one of the cardinal classes described above. However, these types, marked by HB9 and SHOX2, are of interest since they are thought to be candidates for the rhythm-generating neurons (Hinckley et al., 2005, 2010; Wilson et al., 2005, 2007; Hinckley and Ziskind-Conhaim, 2006; Brownstone and Wilson, 2008; Ziskind-Conhaim et al., 2010; Dougherty et al., 2013; Caldeira et al., 2017). The HB9 neurons have a mixed neurotransmitter phenotype and progenitor domain origin. Blocking only glutamatergic transmission had no impact on locomotion, while blocking all synaptic transmission caused defects in the frequency of locomotion but not its left–right or flexor-extensor phase (Caldeira et al., 2017; Koronfel et al., 2021). The SHOX2 population, also known as V2d neurons and partly overlapping with V2a neurons, are ipsilateral excitatory neurons, form recurrent connections, and project to motor neurons (Dougherty et al., 2013; Ha and Dougherty, 2018). Similar to HB9 neurons, silencing them or blocking their transmission affects rhythm, but not pattern, generation (Al-Mosawie et al., 2007; Lundfald et al., 2007; Dougherty et al., 2013). It will be of interest to determine whether these intrinsic rhythm-generating neuron types, which have not been described in fish or frogs, are conserved between vertebrates with less or more varied locomotor demands.

## dI6s inhibitory neurons

Modulation of motor output by dorsal interneurons is a conserved feature across all vertebrates and has typically been associated with the gating of sensory input. In the lamprey, one class of dorsal neuron has been identified: glutamatergic primary sensory neurons, which can be subdivided into touch and pressure cells (Christenson and Grillner, 1988; Fernández-López et al., 2012). In zebrafish and mouse, dorsal interneurons are implicated in sensory-to-motor transmission but are much less well-characterized than ventral neurons. These putative sensory-related populations will be discussed at the end of this review.

**Figure 6:**
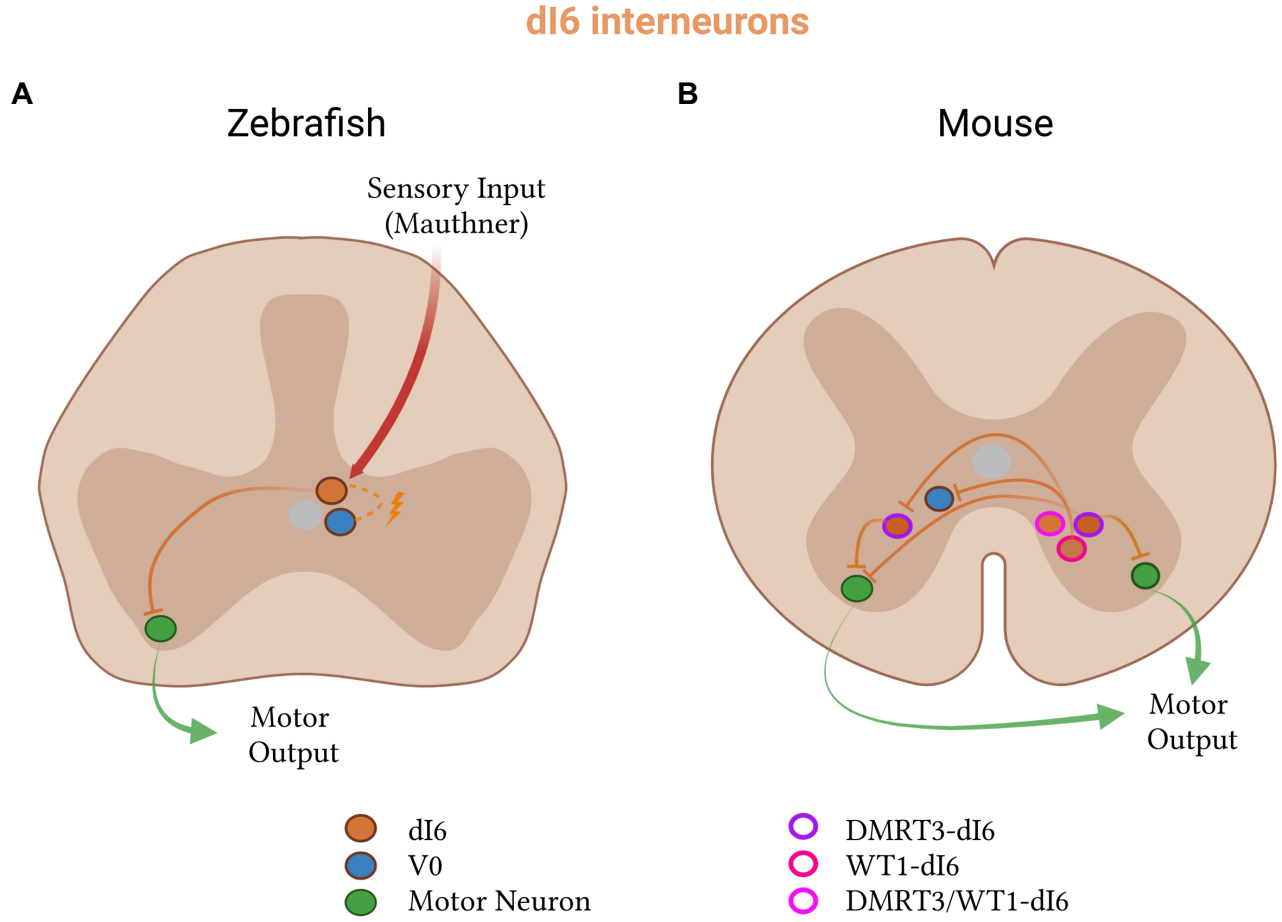
Inhibitory dI6 subtypes in zebrafish and mice. **(A)** In zebrafish, dI6 neurons (orange) receive input from Mauthner cells, form electrical connections with V0 neurons (blue), and project to contralateral motor neurons (green). **(B)** In mice, dI6 neurons split into three subclasses: DMRT3- (purple outline), WT1- (pink outline), and DMRT3- and WT1-co-expressing (pink-red outline). WT1-dI6 inhibit contralateral V0 and DMRT3-dI6 neurons, while DMRT3-dI6 inhibit motor neurons on both sides of the spinal cord.

The exception to this sensory compartmentalization of dorsal interneurons is the inhibitory dI6 population (Figure 6). Like all dorsal populations, the dI6 neurons express LBX1 at early embryonic stages (Gross et al., 2002; Müller et al., 2002). Originating from the dp6 progenitor domain, they additionally express a combination of DBX2 and PAX transcription factors (Andersson et al., 2012; Hernandez-Miranda et al., 2017). The dI6 inhibitory neurons fall into three subtypes based on the expression of DMRT3 and WT1: those that express one, the other or both (Goulding, 2009; Andersson et al., 2012; Schnerwitzki et al., 2018). In zebrafish and mice, dI6 neurons connect to other dI6 neurons and project commissurally to contact motor neurons on the contralateral side to regulate left–right alternation, rhythm generation and locomotor pattern (Soffe et al., 1984; Gross et al., 2002; Müller et al., 2002; Birinyi et al., 2003; Lanuza et al., 2004; Goulding, 2009; Rabe et al., 2009; Dyck et al., 2012; Griener et al., 2017; Haque et al., 2018; Perry et al., 2019; Satou et al., 2020; Uemura et al., 2020).

### Zebrafish dI6 neurons

The dI6 population in zebrafish is inhibitory, expresses Dmrt3a, and has been termed the CoLo (commissural local) neurons based on its anatomical projection pattern (Li et al., 2008; Satou et al., 2009). The function of dI6 neurons seems to vary between developmental stages in zebrafish (Satou et al., 2009).

At escape swimming stages and in the absence of the dI6 population, initiation of the body bend is impaired. This impaired response is specific to Mauthner-mediated escape, in which stimulation of Mauthner cell on one side of the body activates motor neurons on the same side and simultaneously inhibits those on the opposite side (Yasargil and Diamond, 1968). Further studies showed that dI6 neurons are electrically coupled to commissural interneurons and monosynaptically connected to contralateral motor neurons (Figure 6A; Diamond, 1971; Satou et al., 2009). The presence of these connections, together with the altered response of dI6-ablated zebrafish, implies that the dI6 neurons usually function in escape to inhibit the firing of contralateral motor neurons (Satou et al., 2009). Larval dI6 neurons were also found to be inhibited and thus inactive during swimming (Satou et al., 2009), suggesting that their contribution is limited to the Mauthner-mediated escape response at this stage.

At this early swim stage, recent evidence has also linked Dmrt3a-expressing neurons to the regulation of abductor motor neurons in the pectoral fin of zebrafish (Uemura et al., 2020). Abductor and adductor motor neurons alternate in their spiking, like flexor and extensor motor neurons in mammals (Uemura et al., 2020). Abductor, and not adductor, motor neurons receive strong inhibitory synapses from Dmrt3a neurons. In their absence, the timing of abductor neuron firing was impaired, while adductor unaffected. In larval zebrafish, dI6 neurons thus also regulate fin movement via abductor/adductor coordination.

During later-stage beat-and-glide larval swimming, genetic ablation of Dmrt3a led to fewer and shorter movements with decreased velocity and acceleration (Del Pozo et al., 2020). This contrasted with very early coiling stages in which the loss of protein had no effect (Del Pozo et al., 2020), supporting that Dmrt3a-expressing neurons may only be recruited when the fish needs to perform stronger escape movements.

In adult fish, Satou et al. (2020) demonstrated that these neurons were rhythmically active during locomotion, increased their firing probability at slow speeds, and provided mid-cycle inhibition onto contralateral motor neurons. When ablated, there was a decrease in maximum swim speed (Satou et al., 2020). This suggests that dI6 function may change during development: first necessary for strong body bends in larvae and later, required in a speed-dependent manner in adult zebrafish.

### Mouse dI6 neurons

In mice, like zebrafish, a subset of dI6 inhibitory neurons similarly expresses DMRT3. However, expression of WT1, together with GABAergic and glycinergic neurotransmitters, define a broader population of dI6 neurons in the mouse (Figure 6B; Goulding, 2009; Andersson et al., 2012; Haque et al., 2018). This population can be further divided into subtypes based on morphology, electrophysiology, neurotransmitters, birth order, transcription factors and axon guidance gene expression (Andersson et al., 2012; Griener et al., 2017; Schnerwitzki et al., 2018; Perry et al., 2019; Kishore et al., 2020; Iglesias González et al., 2021).

Reinforcing a conserved role of the DMRT3-expressing dI6 subset in cross-body inhibition, *Dmrt3*-null mice exhibit impaired left–right as well as fore-hind limb coordination (Andersson et al., 2012). Other defects include a decrease in swim duration when mice are placed in water, and, when not in water, an increase in twitching movements, an inability to run at high speeds and a decrease in alternation of hindlimb steps during air-stepping. Ablation of *Dmrt3* also led to a dissociation between activity in the contralateral ventral roots, indicating diverse roles in limb coordination (Andersson et al., 2012). Consistent with these behavioral observations, the DMRT3-expressing dI6 neurons in mice are known to contact V1 neurons and motor neurons on both sides of the spinal cord and are rhythmically active during fictive locomotion (Andersson et al., 2012; Griener et al., 2017; Perry et al., 2019). This indicates a conserved role between mice and zebrafish for the DMRT3-dI6 neurons in regulating rhythm and coordinating activity on either side of the spinal cord.

To examine the function of the rest of the dI6 population, Schnerwitzki et al. evaluated *Wt1*-knockout mice (Schnerwitzki et al., 2018). Neonatal mice with this deletion displayed uncoordinated and variable locomotor activity: a slower walk with a decreased stride frequency and increased stride length, and loss of left–right and fore−/hindlimb coordination. The anatomical projection pattern of WT1-dI6 neurons is consistent with these defects. Whereas the only known targets of the DMRT3-dI6 are the motor neurons on both sides of the spinal cord (Andersson et al., 2012), the WT1-dI6 neurons have commissural projections and terminate close to, and likely onto, the DMRT3-dI6 and V0 neurons (Haque et al., 2018; Schnerwitzki et al., 2018). V0 neurons are proposed to excite contralateral inhibitory interneurons, which in turn contact motor neurons (Talpalar et al., 2013; Shevtsova et al., 2016; Danner et al., 2017), conferring an indirect function in contralateral inhibition onto this WT1 population. This indirect function was further supported by the acute silencing of WT1-dI6 neurons (Haque et al., 2018). Acute silencing resulted in the elimination of left–right, but maintenance of flexor-extensor alternation. The group also showed that the bursting of WT1-expressing cells was tightly coupled to fictive locomotor activity of motor neurons. Since WT1-dI6 do not contact motor neurons directly, but instead contact commissural interneuron subtypes, they were thus proposed to indirectly gate the activity of rhythm-generating neurons.

### Cross-species perspective on dI6

Across vertebrates, dI6 neurons play a consistent role in regulating the firing of contralateral motor neurons to coordinate the left and right sides of the body. This role in left–right coordination has recently been shown to be essential for generating the characteristic gaits of horses, with mutations in *Dmrt3* associated with the emergence of new gaits in Icelandic horses (Andersson et al., 2012). Thus, in mice and likely other four-limbed mammals, dI6 neurons have diverged in their molecular, anatomical and functional properties to control same-side inhibition, rhythm and gait generation. These diverse roles in limbed vertebrates seem to map differentially onto the molecularly distinct DMRT3 and WT1 subpopulations. DMRT3-dI6 neurons contact motor neurons directly whereas WT1-dI6 neurons do not. WT1-dI6 neurons instead receive multi-synaptic input and target contralateral interneurons.

These anatomical differences seem to confer each subpopulation with different roles in locomotion in the mouse. Work in horses, mice and zebrafish suggest that the DMRT3-dI6 neurons, with their monosynaptic connections onto motor neurons, are important for the coordination of body bend and gaits, likely via sensory integration (Andersson et al., 2012; Schnerwitzki et al., 2018; Del Pozo et al., 2020) and potentially through flexor-extensor regulation as has been demonstrated in zebrafish (Uemura et al., 2020). WT1-dI6 neurons, in contrast, are proposed to gate the rhythm-generating circuitry in general by integrating supraspinal and proprioceptive input, as well as releasing motor neurons on the opposite side of the spinal cord from same-side inhibition, although it is yet to be shown experimentally whether this is indeed the case (Schnerwitzki et al., 2018).

## Other dorsal interneurons

The dorsal horn of the spinal cord, as the main target area of primary somatosensory afferent axons, is classically implicated in sensory processing of higher order vertebrates (Melzack and Wall, 1965; Todd, 2010, 2017; Abraira and Ginty, 2013; Braz et al., 2014). Our understanding of the interneuron circuitry in the dorsal horn however, is more limited than that of the ventral horn. On a developmental level, we know the molecular determinants of the identity of many classes. On an anatomical and physiological level however, the connectivity and functional properties of these molecularly defined dorsal populations remains unclear.

**Figure 7:**
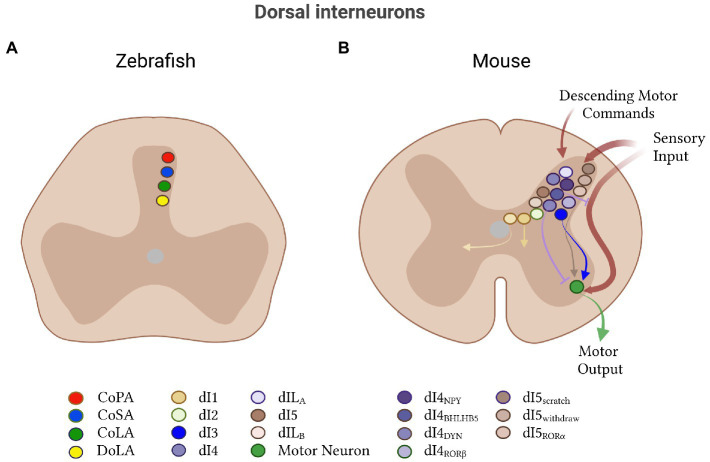
Other dorsal interneurons in zebrafish and mice. **(A)** In zebrafish, four classes of dorsal interneuron have been identified: glutamatergic commissural primary ascending (CoPA, red), glycinergic commissural secondary ascending (CoSA, dark blue), glycinergic commissural longitudinal ascending (CoLA, green), and glycinergic dorsal longitudinal ascending (DoLA, yellow). CoPA neurons drive touch-mediated larval escape. **(B)** In mice, dorsal neurons are divided into seven classes: dI1-5, dIL_A_ and dIL_B_. The glutamatergic dI1 class (yellow) subdivides into ipsi- and contralateral populations. Little is known about the dI2 class (light green). The dI3 class (dark blue) receives cutaneous afferent input and excites motor neurons (green). The dI4 class subdivides according to sensory modality: the NPY subclass (dark purple) is associated with mechanical itch, BHLHB5 (medium-dark purple) with chemical itch, and DYN (medium-light purple) with nociception. RORβ neurons (light purple) gate sensory afferent transmission. They also include dIL_A_ (light purple-pink) and dIL_B_ (light pink-brown) classes. dI5 neurons associated with scratch (dark brown) are located in laminae I/II, and with paw withdrawal reflex (medium brown) in laminae II/III. RORα neurons (light brown) receive descending motor commands and project onto motor neurons, and function in corrective motor adjustments.

In mice, these interneurons project to a variety of targets including supraspinal structures, motor neurons, cutaneous afferents, proprioceptive terminals and other dorsal interneurons (Figure 7A; Helms and Johnson, 2003; Alaynick et al., 2011; Le Pichon and Chesler, 2014; Lai et al., 2016). In lamprey, dorsal interneurons similarly relay and process sensory information, consisting of a glutamatergic dorsomedial, lateral and giant interneuron population (Rovainen, 1967, 1974; Selzer, 1979; Fernández-López et al., 2012). In zebrafish, four dorsal populations have been identified anatomically, including glutamatergic commissural primary ascending (CoPA), glutamatergic and glycinergic commissural secondary ascending (CoSA), glycinergic commissural longitudinal ascending (CoLA), and glycinergic dorsal longitudinal ascending (DoLA) neurons (Hale et al., 2001; Higashijima et al., 2004c). However, a function has only been well-defined for CoPA neurons, which drive early touch-mediated larval escape (Pietri et al., 2009). The lack of extensive characterization or diversity of dorsal neurons in the lamprey, tadpole and zebrafish (Figure 7A; Buchanan and Cohen, 1982; Clarke et al., 1984; Christenson and Grillner, 1988; Li et al., 2004b; Pietri et al., 2009), likely indicates a simplification or absence of some of these populations in aquatic vertebrates – a hypothesis that will likely be tested by molecular cell type profiling in the future.

In all vertebrates, it is well known that sensory inputs can modulate locomotion in a phase-dependent manner (Forssberg et al., 1977; Forssberg, 1979; Andersson and Grillner, 1983; Schillings et al., 1996; Bouyer and Rossignol, 1998). In recent work, an inhibitory population of glycinergic deep dorsal horn parvalbumin-expressing interneurons (dPVs) were found to be active during locomotion, joining the RORβ- and SATB2- expressing populations in representing inhibitory interneurons involved in a cutaneous sensory-motor pathway (Hilde et al., 2016; Koch et al., 2017; Ozeri-Engelhard et al., 2022). The medial deep dorsal horn is an area of large convergence of cutaneous and proprioceptive inputs, and the dPVs integrate these multimodal sensory inputs to modulate cutaneous-evoked muscle inhibition in a state- and phase-dependent manner.

In the mouse, in which dorsal neurons are clearly present in large numbers and arguably best studied, six cardinal classes have been defined based on developmental origin, marker expression, settling position, projection pattern, and neurotransmitter type (Figure 7B; Helms and Johnson, 2003; Lai et al., 2016; Häring et al., 2018; Sathyamurthy et al., 2018; Zeisel et al., 2018). More recently however, an alternative organization has been proposed in which neuron types display a laminar organization that correlates with their function in encoding a specific somatosensory reflex program (Gatto et al., 2021). How this organization links to cardinal class identity is an active area of current study (Le Pichon and Chesler, 2014; Gatto et al., 2021; Russ et al., 2021). Here, we summarize the properties of dorsal interneurons in the mouse in relation to their developmental cardinal class identity: ipsilateral and contralateral dI1, dI2, dI3, dI4, dIL, and dI5.

### dI1/dI2 excitatory neurons

dI1 and dI2 neurons originate from ATOH1- and NEUROG1-expressing progenitors, respectively (Lai et al., 2016). *Atoh1* mouse mutants lack dI1 neurons, extend NEUROG1 expression, and thus generate more dI2 neurons (Gowan et al., 2001). They display minor motor defects (Yuengert et al., 2015), that may result from either the loss of dI1 or gain of dI2 neurons. The dI1 population in mice is composed of an ipsilateral (dI1-ipsi) and a contralateral LHX2-expressing (dI1-contra) population, both of which express the transcription factors BARHL1/2 and are glutamatergic (Wilson et al., 2008; Ding et al., 2012; Lu et al., 2015). During development, *Barhl2* specifies dI1 subtype diversity such that in *Barhl2*-null mice, the dI1-ipsi subpopulation expresses the dI1-contra transcription factor LHX2, and less of the dI1-ipsi-enriched transcription factor BARHL1 (Ding et al., 2012). The dI1-ipsi population in these mice also exhibit a dI1-contra settling and projection pattern, suggesting that BARHL2 is important in specifying dI1 subtype diversity (Ding et al., 2012). Unlike dI1, dI2 interneurons lack BARHL2 and LHX2/9, and instead are characterized by FOXD3, LHX1 and LHX5 expression during development (Avraham et al., 2009; Delile et al., 2019). In mice, very little is known about dI2 anatomy or function. A recent study in chick however, showed that dI2 neurons at limb levels receive sensory and premotor interneuron input, and project to the cerebellum (Haimson et al., 2021). Silencing of dI2 neurons in chick results in abnormal hindlimb stepping (Haimson et al., 2021), implicating them in the high-level coordination of limb movement.

### dI3 excitatory neurons

The dI3 interneuron class is distinguished from other spinal interneurons by the expression of the LIM homeodomain transcription factor ISL1 (Liem et al., 1997; Helms and Johnson, 2003). This class is known to receive direct low-threshold cutaneous afferent input and form excitatory glutamatergic connections with motor neurons and other rhythm-generating interneurons (Bui et al., 2013, 2016). It was demonstrated that the elimination of glutamatergic transmission from these neurons leads to a loss in grip strength in mice, implicating this population in grasping, likely by gating sensory transmission (Bui et al., 2013). Bui et al. suggested that the dI3 neurons are important for functional recovery following spinal cord transection, since their removal had little effect on locomotor activity but a large negative impact on recovery (Bui et al., 2016). They proposed that dI3 neurons compare sensory and locomotor input to compute a prediction error, which could be used to correct locomotor output. It is likely that this is also the mechanism that allows this group of interneurons to produce the appropriate grip force.

### dI4 inhibitory neurons

dI4 interneurons, defined by the expression of PTF1A and GBX1/2 during development (Glasgow et al., 2005; Mizuguchi et al., 2006; Wildner et al., 2006), can be segregated by their birth timing into two subpopulations: the early-born dI4 and late-born dIL_A_ population in mice, which are characterized by the homeodomain factors LHX1/5 and PAX2, respectively (Glasgow et al., 2005; Betley et al., 2009). For both populations, PTF1A is necessary for dI4 interneurons to adopt a GABAergic neurotransmitter profile (Glasgow et al., 2005; Mizuguchi et al., 2006; Wildner et al., 2006). The connectivity and synaptic differentiation of these interneurons is determined by their sensory targets (Betley et al., 2009). In general, silencing dI4 leads to hypersensitivity to mechanical or thermal stimuli and increased pain and itch responses, whereas their activation has the opposite effect (Armbruster et al., 2007; Duan et al., 2014; Foster et al., 2015; Petitjean et al., 2015; Cui et al., 2016; Escalante and Klein, 2020; Mona et al., 2020), directly implicating these populations in the processing of sensory stimuli. Through feed-forward inhibition of motor neurons and presynaptic inhibition of sensory and other interneurons, one such role of dI4 is to filter sensory signals according to the phase of the locomotor cycle (El Manira et al., 1997; Rossignol et al., 2006; Rudomin, 2009; Fink et al., 2014; Goulding et al., 2014). Recent work has also shown that dI4 subtypes segregate according to sensory modality – with those expressing NPY preferentially associated with mechanical itch, BHLHB5 with chemical itch, and DYN with nociception (Ross et al., 2010; Duan et al., 2014; Kardon et al., 2014; Bourane et al., 2015; Koch et al., 2018). Another subset of dI4 neurons expressing RORβ modulate the motor output during walking by gating sensory afferent transmission (Koch et al., 2017).

### dI5 excitatory neurons

LBX1-positive neurons are divided into two populations, one expressing PAX2 (dI4, dI6, and dIL_A_) and inhibitory, and the other TLX3/LMX1B (dI5 and dIL_B_) and excitatory (Gross et al., 2002; Müller et al., 2002; Cheng et al., 2004; Mizuguchi et al., 2006). The dI5 dorsal progenitor domain also produces the excitatory dIL_B_ subset (Gross et al., 2002; Müller et al., 2002). The dI5 interneuron population expressing TLX1/3, LMX1B and ASCL1 (MATH1), conveys information about itch, temperature, static and dynamic touch (Gatto et al., 2019). Ablation of dI5 strongly affects different aspects of somatosensation (Szabo et al., 2015). Recent studies have revealed that sensory modalities map onto spatially, instead of molecularly, distinct dI5 subpopulations in the spinal cord (Gatto et al., 2021). The scratch reflex, for example, is produced by cells in lamina I/II, and the paw withdrawal reflex by those in lamina II/III. Additionally, distinct modules encode low-threshold mechanical stimulation (Abraira et al., 2017), and static and dynamic tactile reflexes, with the latter falling largely into the molecularly distinct RORα subpopulation (Bice and Beal, 1997a,b; Spike et al., 2003; Cheng et al., 2005; Yasaka et al., 2010; Gatto et al., 2021). This population receives descending motor commands and projects onto motor neurons. It is a key part of the spinal touch circuitry that underlies corrective motor adjustments (Bourane et al., 2015).

In summary, most of the work on dorsal populations has been carried out in mice. Due to the multi-modal nature of sensory perception and integration in the mouse as compared to the fish, it is thus not surprising that current evidence supports that the dorsal interneuron populations of mice are more numerous and exhibit much greater heterogeneity than in simpler vertebrates. This hypothesis that the dorsal spinal cord expanded and diversified over vertebrate evolution can be addressed in the future with high-throughput molecular and physiological techniques that are increasingly becoming feasible in non-mammalian and less characterized vertebrates.

## Discussion

Our comparison of interneurons across vertebrates finds several potent examples of neuron-to-function conservation, but just as many of new neural classes and subtypes that emerge with the more muscle groups and complex movement patterns of higher order species. Accordingly, the division of existing cardinal classes into multiple subclasses appears to be a prevalent theme going from lamprey to zebrafish to mice and more broadly, from swimming-to-limb-based movement.

### Conservation of interneurons across vertebrates

One common theme to all vertebrate spinal circuits is the conservation of the three-part basic spinal rhythm-generating circuits beginning with the most primitive extant vertebrate, the lamprey. This architecture of motor neurons, ipsilateral V2a-type excitatory neurons, and commissural V0-type inhibitory neurons is present in the lamprey, tadpole, zebrafish and mouse spinal cord. In addition, zebrafish and mice have all ventral cardinal classes including not only the V2a (Figure 2) and V0 (Figure 4), but also the excitatory V3 (Figure 3) as well as the inhibitory V1 and V2b (Figure 5), and dorsal dI6 (Figure 6) populations.

We also observe functional conservation between finned and limbed vertebrates, with subclasses for left–right and rostral-caudal coordination, as well as those for graded muscle recruitment with increasing drive, common to both. In zebrafish and mice, for example, the excitatory V2a, V0, and inhibitory V1/V2b neurons are composed of multiple speed-specific subtypes, thus controlling the frequency of locomotion. The V0v and V0d subpopulations are important for coordinating diagonal activity and providing mid-cycle inhibition in both species. These subclasses are thus responsible for pan-vertebrate features such as speed-dependent recruitment of motor neurons, and coordination along and across the body axis.

### Species-specific interneuron subpopulations

Between vertebrate species that swim versus walk however, there are notable differences. In some cases, existing neuron types for swimming seem to have taken on new roles in limb-based movement. For example, V1 and V2b inhibitory neurons, in addition to coordinating basic features of locomotion across vertebrates such as the frequency of movement, also regulate flexor-extensor alternation in limbed vertebrates. The dI6 class, necessary for the escape response and mid-cycle inhibition to motor neurons in zebrafish, also controls gaits in limbed organisms. The V0 classes have additionally taken on the role of speed-dependent left–right coordination in mice.

From this literature review, it is also clear that the more complex and variable the movement patterns and gaits of a vertebrate, the more their interneurons have been compartmentalized into distinct subtypes. This subdivision is based on factors such as birth date, projection range, physiology, recruitment threshold and function. Exemplifying this, the V2a population in mice is split into one subpopulation that receives locomotor drive and one that does not. These subpopulations differ in their marker expression and projection patterns along the rostrocaudal axis of the spinal cord. This diversity is directly linked to greater dexterity of the forelimbs. In the same manner, the subdivision of the V1 class in mice into around 50 molecularly distinct types is likely to have allowed the class to take on new functions in flexor-extensor control, mediated by Ia- and Ib-inhibition (Benito-Gonzalez and Alvarez, 2012; Britz et al., 2015; Bikoff et al., 2016; Gabitto et al., 2016; Sweeney et al., 2018). The V3 population, which seems to contribute to excitatory drive in fish and mice, can be split into subpopulations in mice, which have not been found in zebrafish, and are necessary for the generation of the trotting gait (Borowska et al., 2013; Chopek et al., 2018; Deska-Gauthier et al., 2020; Zhang et al., 2022). These subdivisions allow them to control the balance of activity on both sides of the spinal cord more precisely, essential for locomotion on land.

Entirely new cardinal classes of interneurons also seem to have developed to facilitate limbed locomotion. The V0c and V2c subclasses were found in mice, homologues of which have not been discovered in zebrafish. Many new sensory dorsal interneuron classes are additionally present in mice but have not been identified in lamprey, zebrafish, or tadpoles, possibly enabling specific features of locomotion in terrestrial sensory environment. Dorsal neurons in mice are molecularly heterogeneous and have overlapping but sensory-specific functions (Gatto et al., 2019). From work in the cat, it has become clear that sensory inputs are important for initiating and maintaining an appropriate locomotor rhythm by regulating phase changes during stepping and modulating the amplitude of motor output (Stuart and Hultborn, 2008). Dorsal interneurons also project supraspinally and receive descending input to regulate descending pathways (Bourane et al., 2015; Haimson et al., 2021). Since dorsal spinal interneurons regulate these sensory pathways, one can be certain that they will exhibit large differences between water- and land-based animals, consistent with current observations of dorsal subtype radiation from zebrafish to mouse (Figure 7). The specific differences at a molecular, anatomical and functional level will be an interesting area to examine in the future.

Location, connectivity, proportion and number of neurons of each class are also important parameters that can change the output of a neuronal circuit in a species-specific manner. The same class could be present in two species, but its function could differ. Exemplifying this, some classes can be subdivided differentially between species based on the location of their cell body. For the V1 population, this location can be important to enable precise connectivity between interneurons and motor neurons, for example Bikoff et al. (2016). The grouping of V2a interneurons, based on rostrocaudal location, also exemplifies this principle. As has been best shown in the turtle, there are also large differences in distribution between interneurons connected to functionally distinct motor pools (Goetz et al., 2015). Premotor interneurons that project to axial muscles are distributed symmetrically on either side of the spinal cord, while those that connect to limb motor neurons are mainly ipsilateral. These interneuron subpopulations can be distinguished by their genetic profile and neurotransmitter identity. On the other hand, an interspersed distribution of interneuron subtypes that are recruited at different speeds can allow smooth transitions between speeds, as exemplified by the well-defined slow, intermediate and fast circuits of zebrafish (Berg et al., 2018).

Changes in connectivity between neuron classes are likely to have also been necessary for producing more complex movement patterns. Supraspinal connectivity of the cervical V2a subtypes in mice enables precise motor control of the forelimbs (Hayashi et al., 2018). Inter-connectivity of interneurons may also be important for relaying, gating and distributing motor commands. Recent studies have also highlighted that interneurons subdivide based on whether they project locally or long-range (Osseward et al., 2021). V3 neurons, for example, are divided into a local and an ascending population that is important for trot in mice (Zhang et al., 2022). The V2a class, in addition to their role in regulating flexor-extensor activity, has long-range V2a and V2b neurons that are important for ipsilateral body coordination (Hayashi et al., 2023). In limbed vertebrates, that require coordination at and across highly variant regions of the body, it is likely that more examples of such local and long-range divisions with distinct functions will be found for other classes in the future. This may be especially important for limbed vertebrates with high dexterity, such as mice and humans, and movements that require intricate limb-torso or inter-limb coordination, such as trotting in horses.

However, it is important to consider that similar locomotor outputs can be generated with different circuit connectivity. This has been demonstrated in the crab stomatogastric system, and two related species of nudibranchs, and has been proposed to ensure robust circuit function given individual variability (Marder et al., 2015; Sakurai and Katz, 2017). This suggests that there could be some flexibility in how spinal circuits connect to generate a movement pattern.

It is also still unclear to what extent the diversity of spinal neuron types in limbed vertebrates scaled with evolutionary time. Spinal neuron-type diversity increased with the complexification of the body plan and the control of limbs, as we have summarized here. Yet some of the core elements needed for limb control, such as flexor and extensor limb motor neurons and Ia-inhibitory interneurons, are likely to already present in skates and sharks (D’Elia and Dasen, 2018; Jung et al., 2018). Therefore, these elements may have existed in the ancestor of all vertebrates with paired appendages, not just those with limbs. Future molecular and functional studies across even more finned and limbed vertebrates will be crucial in addressing this question.

### Beyond interneurons

Although we have chosen not to focus on motor neurons in this review, it is now clear that they can also influence the pattern and frequency of locomotion, as well as connectivity with premotor networks (Goetz et al., 2015; Hinckley et al., 2015; Baek et al., 2017). In zebrafish, this is mediated by gap junctions, which allow motor neurons to exert influence over the strength of excitation retrogradely (Song et al., 2016), whereas in mice, this is mediated by synaptic glutamate release (Mentis et al., 2005; Nishimaru et al., 2005). Additionally, motor neurons receive strong input from each other, with the fast-type receiving greater excitation than the slow-type (Bhumbra and Beato, 2018).

The traditional concept of interneuron subtype-based locomotor pattern generation is now also being brought into question by work in the turtle. The spinal networks for swimming and scratching movements in turtles largely overlap (Berkowitz, 2002; Hao et al., 2011). The identification of spinal neurons in the turtle is still much less advanced than in the mouse. However, it is clear that the interneurons active during swimming and multiple forms of scratching likely contribute directly to motor output, while the interneurons specialized for one behavior receive hyperpolarizing inhibition during the others (Berkowitz, 2005, 2007, 2008). Additionally, recordings from the lumbar spinal cord of the turtle demonstrate that the neural circuitry exhibits “rotational dynamics,” rather than alternating activity as proposed in the half-center model (Lindén et al., 2022). These observations, although not inconsistent with the diversity of interneurons present in the spinal cord, propose a new way of thinking about pattern-generation as a neural network, as opposed to neural subtype, property.

### Computational dissection of neuron-to-behavior relationships across species

**Figure 8:**
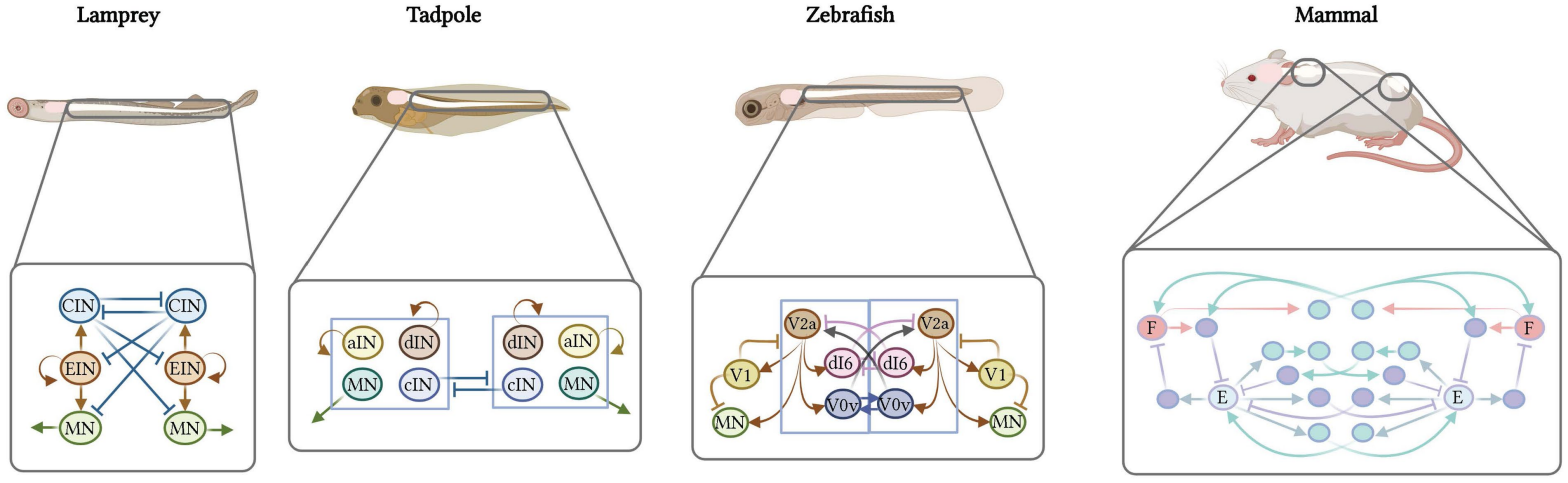
Computational models of the lamprey, tadpole, zebrafish and mammal spinal cord networks. The region of the spinal cord modeled is indicated. Far left: lamprey CPG with excitatory interneurons (EINs), inhibitory commissural interneurons (CINs), and motor neurons (MNs). Middle left: CPG model of the tadpole with the same neuron types as the lamprey plus additional ipsilateral inhibitory neurons (aINs). Middle right: CPG of the zebrafish larva at a stage when it can perform beat-and-glide swimming. Compared to the tadpole model, this model has additional contralateral excitatory neurons. Far right: model of the mammalian spinal cord showing connections between right and left rhythm generators (red: flexor unit, F; blue: extensor unit, E) at the limb level. Turquoise circles represent excitatory interneurons while purple circles represent inhibitory interneurons.

Computational models have emerged as a useful tool to guide our understanding of the function of interneurons in rhythm generation across species (Figure 8; Traven et al., 1993; Ekeberg and Grillner, 1999; Huss, 2007; Ijspeert et al., 2007; Kozlov et al., 2007, 2009; Harischandra et al., 2011; Knüsel et al., 2013; Sarvestani et al., 2013; Roberts et al., 2014; Rybak et al., 2015; Shevtsova et al., 2016; Borisyuk et al., 2017; Danner et al., 2017, 2019; Koutsikou et al., 2018; Ferrario et al., 2021; Zhang et al., 2022), and can help us to address some of the questions raised in this review, namely whether movement complexity parallels neuronal complexity. They can explain experimental observations, by testing the minimal requirements to reproduce these observations *in silico*. They can also help generate hypotheses about how neuronal components can be expected to change their configuration to produce varying output across development, stimuli, or species.

In zebrafish, CPG models have provided a potent demonstration of how the addition of interneuron classes can lead to increasingly complex motor patterns during development (Roussel et al., 2021). Three models were built to test the neuronal basis of the zebrafish’s transition from single-coiling to double-coiling to beat-and-glide swimming. The first model generated single coiling with three components: a pacemaker kernel, V0d-equivalent interneurons, and motor neurons. The addition of V2a and V0v-equivalent neurons, and new chemical synapses, in the second model resulted in the emergence of double coiling. Finally, in the third model, the division of the circuitry into network oscillators, the addition of V1-equivalent interneurons, and a change to mainly chemical synapses gave rise to beat-and-glide swimming. The transition from simple to more complex architecture in these models supports the hypothesis that movement complexity requires increased neuronal and synapse heterogeneity, and generates testable predictions of which neuronal components are required at each stage.

Spinal cord models have also provided a foundation for dissecting how sensory feedback shapes movement. In lamprey and salamander, they have underlined the importance of such feedback in gait transitions and action selection, showing that changes in descending input upon sensory stimulation produced variant motor output (Traven et al., 1993; Ekeberg and Grillner, 1999; Ijspeert et al., 2007; Harischandra et al., 2011; Knüsel et al., 2013; Sarvestani et al., 2013; Koutsikou et al., 2018). In tadpole, they have shed light on the biophysical properties of the spinal circuits that control time-delays in swimming in response to sensory stimulation (Roberts et al., 2014; Borisyuk et al., 2017; Ferrario et al., 2021).

CPG models have also assigned a function, or lack thereof, to anatomically- or molecularly-defined neuron subtypes. It was previously believed, for example, that ipsilateral inhibitory interneurons formed a key part of the rhythm-generating circuitry in the lamprey. However, biophysical models demonstrated ipsilateral excitatory and commissural inhibitory, but not ipsilateral inhibitory, neurons were required (Huss, 2007; Kozlov et al., 2007, 2009). The maintenance of rhythm in the absence of ipsilateral inhibition resulted in a major revision to our understanding of the cellular basis of lamprey locomotion.

Comparing across species, models of the mammalian CPG are much more complex in their neuronal composition and connectivity than those of the lamprey, tadpole or salamander. The most recent mammalian CPG model consists of 12 interneuron types, as opposed to the two in the lamprey (Huss, 2007; Kozlov et al., 2009; Zhang et al., 2022). Importantly, the CPG models can be, and are periodically, updated based on new experimental evidence (Rybak et al., 2006; Shevtsova et al., 2016; Danner et al., 2017, 2019; Zhang et al., 2022), helping to integrate new data into the framework of our current understanding. The most recent update incorporates newly identify lumbar V3 neurons with ascending projections to cervical areas (Zhang et al., 2022). Experimental evidence shows silencing the entire V3 population led to instability of the trotting gait. However, only with the updated model can this defect be localized to the ascending V3 population.

CPG models also provide a means to translate between different forms of pattern generation, such as breathing and locomotion (Garcia-Campmany et al., 2010; Smith et al., 2013; Del Negro et al., 2019), thus yielding a generalizable understanding of variation in rhythmic circuits. Moving forward, they provide a powerful means to probe the cellular basis of locomotor variation within and across species.

## Concluding remarks

Cross-species comparisons *in vivo* and *in silico* can drive observations and predictions about the diversity and function of spinal interneuron types in relation to movement. From this review and its comparison of vertebrate motor circuits, we observe that heterogeneity within a class correlates with finer-tuned control of muscles and a greater movement repertoire. This heterogeneity gives rise to a variety of circuit-level features that facilitate limbed, as opposed to swim, locomotion. Recent development of new tools to examine lesser-studied vertebrates along the swim-to-limb evolutionary trajectory will bring us closer to identify their spinal cords and the remarkable symphony of interneurons across species.

## Author contributions

AW wrote the first draft and prepared the figures. LS revised the review and figures, together with AW. All authors contributed to the article and approved the submitted version.

## Funding

This work was supported by the ERC Starting grant, ERC-2021-STG #101041551.

## Conflict of interest

The authors declare that the research was conducted in the absence of any commercial or financial relationships that could be construed as a potential conflict of interest.

## Publisher’s note

All claims expressed in this article are solely those of the authors and do not necessarily represent those of their affiliated organizations, or those of the publisher, the editors and the reviewers. Any product that may be evaluated in this article, or claim that may be made by its manufacturer, is not guaranteed or endorsed by the publisher.
